# Macromolecular crowding links ribosomal protein gene dosage to growth rate in *Vibrio cholerae*

**DOI:** 10.1186/s12915-020-00777-5

**Published:** 2020-04-29

**Authors:** Alfonso Soler-Bistué, Sebastián Aguilar-Pierlé, Marc Garcia-Garcerá, Marie-Eve Val, Odile Sismeiro, Hugo Varet, Rodrigo Sieira, Evelyne Krin, Ole Skovgaard, Diego J. Comerci, Eduardo P. C. Rocha, Didier Mazel

**Affiliations:** 1 Institut Pasteur, Unité Plasticité du Génome Bactérien, UMR3525, CNRS, Paris, France; 2 Instituto de Investigaciones Biotecnológicas “Dr. Rodolfo A. Ugalde,” CONICET - Universidad Nacional de San Martín, San Martín, Buenos Aires, Argentina; 3 Microbial Evolutionary Genomics, Département Génomes et Génétique, Institut Pasteur, Paris, France; 4 Centre National de la Recherche Scientifique UMR3525, Paris, France; 5 Department of Fundamental Microbiology, University of Lausanne, Quartier SORGE, 1003 Lausanne, Switzerland; 6 Institut Pasteur, Plate-forme Transcriptome et Épigenome, Biomics, Centre d’Innovation et Recherche Technologique (Citech), Paris, France; 7 Fundación Instituto Leloir, IIBBA-CONICET, Buenos Aires, Argentina; 8 Department of Science and Environment, Roskilde University, Roskilde, Denmark

**Keywords:** Ribosomal proteins, Macromolecular crowding, Growth rate, *Vibrio cholerae*, Bacterial chromosome, Bacterial physiology, Synthetic biology

## Abstract

**Background:**

In fast-growing bacteria, the genomic location of ribosomal protein (RP) genes is biased towards the replication origin (*oriC*). This trait allows optimizing their expression during exponential phase since *oriC* neighboring regions are in higher dose due to multifork replication. Relocation of *s10-spc-α* locus (S10), which codes for most of the RP, to ectopic genomic positions shows that its relative distance to the *oriC* correlates to a reduction on its dosage, its expression, and bacterial growth rate. However, a mechanism linking S10 dosage to cell physiology has still not been determined.

**Results:**

We hypothesized that S10 dosage perturbations impact protein synthesis capacity. Strikingly, we observed that in *Vibrio cholerae*, protein production capacity was independent of S10 position. Deep sequencing revealed that S10 relocation altered chromosomal replication dynamics and genome-wide transcription. Such changes increased as a function of *oriC*-S10 distance. Since RP constitutes a large proportion of cell mass, lower S10 dosage could lead to changes in macromolecular crowding, impacting cell physiology. Accordingly, cytoplasm fluidity was higher in mutants where S10 is most distant from *oriC*. In hyperosmotic conditions, when crowding differences are minimized, the growth rate and replication dynamics were highly alleviated in these strains.

**Conclusions:**

The genomic location of RP genes ensures its optimal dosage. However, besides of its essential function in translation, their genomic position sustains an optimal macromolecular crowding essential for maximizing growth. Hence, this could be another mechanism coordinating DNA replication to bacterial growth.

## Background

**Fig. 1:**
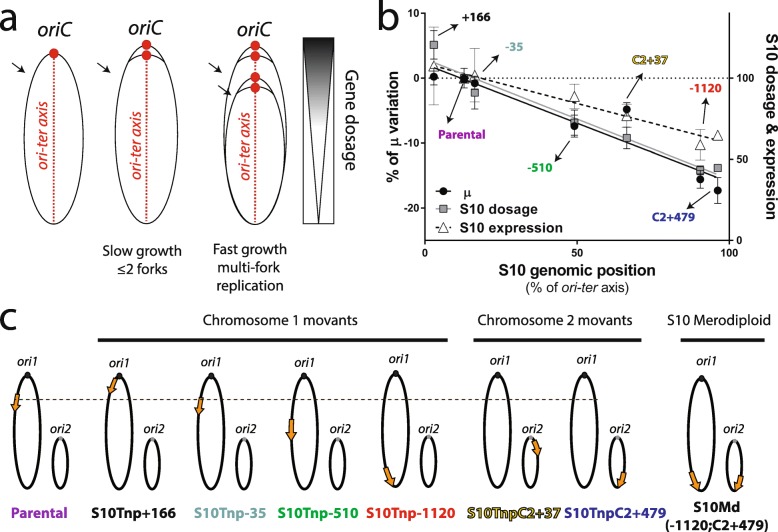
S10 location impacts cell physiology. **a** General bacterial genome structure: the *oriC* (red dot) determines two symmetric replichores along the *ori-ter* axis (left panel). When bacteria grow slowly, genes have 1 to 2 copies (center). During exponential growth, fast growers overlap replication rounds increasing the dosage of *oriC*-neighboring regions (right panel). The approximate position of the S10 locus is shown by an arrow. **b** The maximum growth rate (μ) and the relative S10 dosage and expression with respect to the parental strain plotted as a function of S10 position along the *ori-ter* axis within *V. cholerae* genome. **c** Genome structure of the parental, the movant, and the merodiploid strains employed in this study. The orange arrow represents S10 displaying its genomic position and ploidy. The dashed line represents the S10 location in the parental strain. Chromosomes are drawn according to their replication timing

Replication, gene expression, and segregation are tightly coordinated with the cell cycle to preserve cellular homeostasis [1, 2]. Genome structure may contribute to integrate these many simultaneous processes occurring on the same template. Their relative simplicity and the increasing amount of available data render bacterial genomes ideal models to study this subject [3–6]. Bacterial chromosomes are highly variable in their gene content, but highly conserved in terms of the order of core genes in the chromosomes. Replication begins at a sole replication origin (*oriC*), proceeding bidirectionally along two equally sized replichores until the terminal region (*ter*). This organizes the genome along an *ori-ter* axis that interplays with cell physiology (Fig. 1a) [4, 5, 7]. For instance, essential genes are overrepresented in the replicative leading strand to avoid head-on collisions between the replication and transcription machineries [8]. Large inversions occur preferentially symmetrically with respect to the *ori-ter* axis to avoid the emergence of replichore size imbalance [9, 10]. Recent studies indicate that gene order within the chromosome may play a relevant role in harmonizing the genome structure with cell physiology. Remarkably, key genes coding for nucleoid-associated proteins, RNA polymerase modulators, topoisomerases, and energy production are arranged along the *ori-ter* axis following the temporal order of their expression during growth phases [11, 12]. In addition, recent studies have showcased an increasing number of traits whose expression is influenced by the genomic position of its encoding genes [13–18]. Notable examples are genes encoding the flux of the genetic information. In fast-growing bacteria, the genes coding for transcription and translation machineries locate near the *oriC* [19, 20]. These microorganisms divide faster than the time required for genome duplication. Consequently, chromosomes trigger replication more than once before cytokinesis, overlapping successive DNA duplication rounds, a phenomenon called multifork replication (Fig. 1a). This leads to replication-associated gene dosage gradients along the *ori-ter* axis during exponential growth (Fig. 1a) [14]. Therefore, it was proposed that the *oriC*-proximal location of ribosomal and transcription genes allows the recruitment of multifork replication for growth optimization purposes [5, 19, 20]. Thus, the dosage and expression of the aforementioned genes peak during exponential growth phase (Fig. 1a, right) when the transcriptional activity and ribosome numbers increase by 10- and 15-fold, respectively [21].

In previous works [22, 23], we tackled this issue in *Vibrio cholerae*, the causative agent of cholera disease. This bacterium harbors a main chromosome (Chr1) of 2.96 Mbp and a 1.07-Mbp secondary replicon (Chr2). Their replication is coordinated since the *oriC* of Chr2 (*ori2*) fires after 2/3 of Chr1 duplication has elapsed, finishing the process synchronously [24, 25]. *V. cholerae* is among the fastest-growing bacteria displaying particularly high replication-associated gene dosage effects [19]. Its transcription and translation genes map close to the *oriC* of Chr1 (*ori1*) [22]. Among them, *s10-spc-α* (S10) is a 13.4-Kbp locus harboring half of the ribosomal protein genes (RP) located 0.19 Mbp away from *ori1* [22]. Using recombineering techniques, we built a set of S10 *movants* (i.e., isogenic strains where the genomic position of S10 locus is modified) to uncover interplays between the chromosomal position of the locus and cell physiology. We found that its growth rate decreased as a function of the distance between S10 and *ori1* (Fig. 1b, c). Also, S10 genomic location impacted on *V. cholerae* fitness and infectivity [22, 23]. As predicted by bioinformatics [19, 20], we showed that *oriC* proximity of S10 provides optimal dosage to attain the maximal growth capacity [22]. But we also found that S10 position impacts bacterial fitness in the absence of multifork replication suggesting that the RP gene location affects cell physiology even in slow-growing bacteria [23]. In sum, our previous work and the cited examples [14] support the notion that gene order conditions cell physiology, shaping genome structure along the evolution.

Although we proved that the current S10 genomic location maximizes *V. cholerae* fitness [22, 23], we still lack a mechanism explaining this phenomenon. Here, we addressed this issue through the most straightforward hypothesis that is S10 relocation far away from *ori1* diminishes ribosome component availability. This, in turn, should reduce ribosomal activity, impacting cell physiology globally through the general impairment of protein synthesis. In this work, we quantified the global protein production in the parental strain and in the most affected derivatives (Fig. 1b, c). Surprisingly, we found no differences in global protein production. RNA and DNA deep sequencing revealed genome-wide alterations in gene transcription and replication dynamics suggesting the existence of global mechanisms linking S10 dosage to cell physiology not linked to protein biosynthesis capacity.

The intracellular milieu has a very high concentration of macromolecules that reaches 400 mg/mL in *Escherichia coli*. Consequently, the cytoplasm does not behave as an ideal solution since this large quantity of macromolecules occupies 20–30% of its volume, which is physically unavailable to other molecules. Such steric exclusion creates considerable energetic consequences, deeply impacting intracellular biochemical reactions. This phenomenon, referred to as macromolecular crowding [26, 27], has received little attention in in vivo systems [28, 29]. Protein accounts for ~ 55% of the bacterial cell mass [21, 26], with RP representing one third of them [30]. We hypothesized that S10 expression reduction would lead to lower macromolecular crowding within the bacterial cytoplasm, globally affecting cell physiology [26, 28, 29]. Here, we gathered evidence supporting the idea that S10 relocation mainly impacts cellular physiology of *V. cholerae* by altering cytoplasm homeocrowding (i.e., macromolecular crowding homeostasis) [26].

## Results

### S10 relocation does not cause ribosomal activity reduction in normally growing cells

**Fig. 2:**
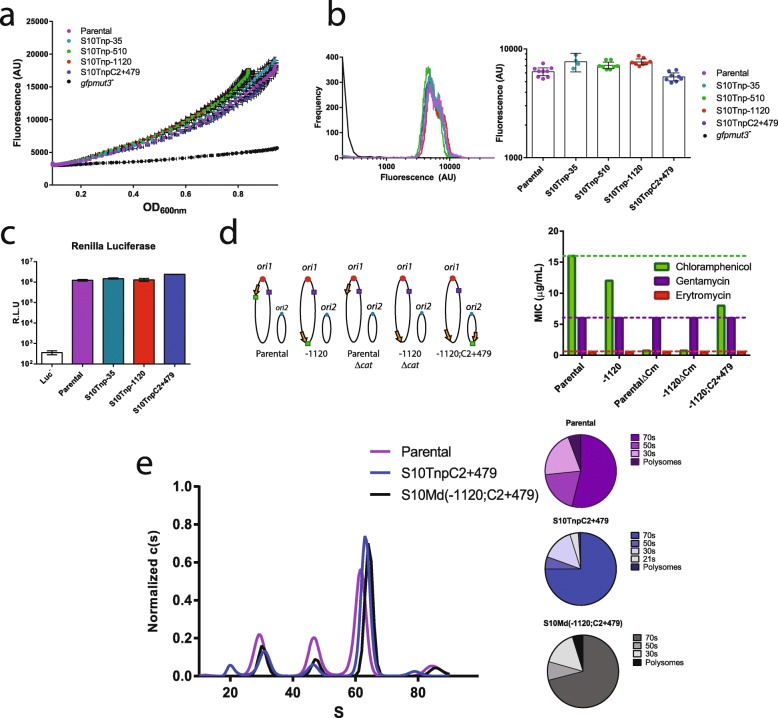
S10 genomic location does not impact ribosome function in normally growing cells. **a** The GFP expression and OD_600nm_ of the indicated *gfpmut3*^+^ strains were measured along time. The fluorescence mean (± SD) was plotted as a function of the mean (± SD) OD_600nm_. Figure shows a representative of 3 independent experiments with 4 biological replicates. The parental *gfpmut3*^*−*^ strain is an autoflourescence/light dispersion control. **b** The indicated *gfpmut3*^+^ strains in early exponential phase were analyzed by FC. Left panel shows the fluorescence signal frequency distribution of the indicated *V. cholerae* strains. A *gfpmut3*^*−*^ strain was added as negative control. Right panel shows the fluorescence intensity with the 95% confidence interval (CI). Points represent individual biological replicates obtained along at least 2 independent experiments. **c** Parental and movant strains bearing RLU in the chromosome (Table S1) were grown until early exponential phase. Then, RL activity, represented as RL units (RLU), was measured in three independent biological replicates for each strain. **d** Parental and derivative strains present similar resistance levels to ribosome-targeted antibiotics. On the right panel, chromosomes are represented as in the previous figure. The encoded antibiotic resistance markers are depicted as boxes: Gm in violet and Cm in green. Their approximate genomic location is shown in each strain. On the right, the MIC (μg/mL) for Cm, Gm, and Er for each depicted strain is shown. **e** Ribosome profiles for the indicated strains as obtained by AUC. Pie charts quantify polysome, 70s, 50s, and 30 s fractions for the indicated strains

S10 relocation impacts cell physiology in a dosage-dependent manner [22, 23]. However, how S10 dosage reduction affects cell physiology is still unknown. The most straightforward explanation is that a reduction of RP levels upon S10 locus relocation affects ribosome biogenesis leading to a reduction in protein synthesis. To inquire if S10 relocation impairs protein production, we created strains expressing GFP under a strong constitutive promoter into an innocuous intergenic space (Additional File 1: Table S1). The direct quantification of fluorescence allows for estimation of protein production capacity in each strain [31]. First, we followed in time the optical density (OD) and the fluorescence signal of these derivatives. We estimated translation capacity by plotting fluorescence as a function of OD (Fig. 2a). Fluorescence increased exponentially as the OD incremented (*R*^2^ > 0.99, Additional File 1: Table S2). Although the curves differed slightly between strains, there was no significant correlation between S10 genomic position and GFP production (Pearson’s test, *r* = 0.1, *p* = 0.86). We next subjected cultures of these strains to flow cytometry during early exponential phase, when S10 dosage differences among the movants are maximal. This method allows to simultaneously observe the average GFP production per cell with higher sensitivity and the distribution of fluorescence among the cells in the populations (Fig. 2b). All tested strains showed similar signal levels and the same distribution pattern. In sum, we found no link between GFP production and S10 genomic location.

To confirm that these results were not due to lack of sensitivity, we used the *Renilla* luciferase (RL) as a reporter of protein synthesis capacity. RL detection shows higher sensitivity than GFP due to lower background, higher signal amplification, and a larger dynamic range, making it suitable to reveal more subtle differences otherwise impossible to differentiate [32]. We built S10 movant strains constitutively expressing RL at high levels (Additional File 1: Table S1). Again, no differences in the luciferase activity arose between the parental strain, S10Tnp-35, S10Tnp-1120, and S10TnpC2+479 (Fig. 2c), suggesting similar translation capacity.

It was recently reported that reduction in the number of ribosomes increases the sensitivity to ribosome-targeted antibiotics [33]. Hence, we measured the minimum inhibitory concentration (MIC) of chloramphenicol (Cm), gentamicin (Gm), and erythromycin (Er) in the parental and movant strains (Fig. 2d). All the tested mutants derive from a *V. cholerae* isolate sensitive to Er and harboring Gm resistance gene (Additional File 1: Table S1). Strains that only differed in the genomic location of S10 had their growth inhibited at the same Er and Gm concentrations (Fig. 2d) suggesting no differences in ribosomal numbers. In parallel, the parental, S10Tnp-1120, and S10Md(-1120;C2+479) strains harbor the Cm resistance gene (*cat*) linked to the S10 locus; therefore, the location of the resistance gene differed among them (Fig. 2d). Cm resistance was higher in the parental strain when *cat* is closer to the *ori1* and lower in S10Tnp-1120 and S10Md(-1120;C2+479) when the resistance marker is nearby the *ter1* region. Hence, as in other genetic systems [34], Cm sensitivity varied according to *cat* genomic location independently of S10 copy number (compare S10Tnp-1120 to S10Md(-1120;C2+479)). Therefore, even though this assay is sensitive enough to capture the effects caused by differences in *cat* genomic location, it showed no antibiotic susceptibility differences related to S10 genomic location. The lack of effects of S10 relocation on MIC when using any of the three different ribosome-targeting antibiotics, possessing different tolerance levels, suggests that the number of ribosomes is not affected by the genomic location of S10.

### S10 genomic location causes changes in maximum GFP synthesis capacity

Since we did not detect differences in the ribosomal activity with previous approaches, we measured GFP production at the single cell level using fluorescence recovery after photobleaching (FRAP). In this assay, the full area of individual cells expressing GFP was photobleached and followed over time for at least 5 min. Then, we quantified the percentage of fluorescence recovery. This allows comparing the maximum capacity of protein synthesis between strains. In the parental strain, ~ 95% of the cells displayed a recovery of at least 20% (mean = 53.8%, *n* = 108) of the initial signal after 3 min, to reach a plateau until the end of the observation (Additional File 1: Fig. S1a). The addition of Cm up to the MIC significantly inhibited the fluorescence increase (mean = 15.8%, *n* = 21, *p* < 0.0001), suggesting that signal recovery corresponds to GFP re-synthesis. Meanwhile, we observed a significantly lower average recovery in the most physiologically affected movants S10Tnp-1120 (20.1%, *n* = 42, *p* < 0.0001) and S10TnpC2+479 (25.8%, *n* = 82, *p* < 0.0001) (Additional File 1: Fig. S1b), suggesting that they produced less GFP. Therefore, the parental strain displayed a higher maximum protein synthesis capacity than the most affected S10 movants.

### S10 relocation alters the ribosomal sedimentation profile

Reduction in RP expression can lead to problems in ribosome assembly due to modifications in the stoichiometry of its components. To detect alterations in ribosome assembly, reflected in changes in ribosomal subunits composition, we performed ribosome preparations followed by analytical ultracentrifugation (AUC) in the parental and the physiologically impaired S10TnpC2+479 strain. We also analyzed a merodiploid strain where most of the growth deficiency is rescued but still displays a reduced growth (S10Md(-1120;C2+479)) [22]. We expected that growth impairment would correlate with a reduction in the proportion of assembled ribosomes (i.e., the 70s peak), when compared to free ribosomal subunits (30s and 50s peaks). Figure 2e shows that parental strain displayed a 53.97% of the signal in the peak corresponding to the 70s while 50s and 30s peaks represented 19.4 and 20.8%, respectively. In the S10TnpC2+479 movant, we observed an increase in the 70s proportion to the 75.85% of the signal while the free ribosomal subunits lowered to 5.5% and 14.8% of the signal for 50 and 30s subunits, respectively. In the S10Md(-1120;C2+479) strain, showing an intermediate growth phenotype, 70s, 50s, and 30s represented 71%, 8.3%, and 15.8% of the signal, respectively. Our data shows that a reduction in S10 expression led to an increase of the proportion of assembled ribosomes and a reduction of free ribosomal subunits. Therefore, movant strains might compensate lower S10 expression engaging more free ribosomal subunits into translation. This could explain the relatively low impact of S10 relocation on translation capacity.

### Dosage reduction of S10 non-ribosomal genes does not impact cell physiology

Since reduction of protein biosynthesis upon S10 relocation was mild, we reasoned that it cannot explain the drastic changes observed in fitness and growth rate (μ). Meanwhile, S10 harbors genes not related to ribosome biogenesis: *rpoA*, encoding for the α-subunit of RNA polymerase, and *secY*, which codes part of the Sec translocon [35], essential for protein export. We wondered whether dosage reduction of *rpoA* and/or *secY* could contribute to the phenotype caused by S10 relocation by provoking a reduction of the transcription rate and/or by hampering the normal protein export process. To test this, we cloned *rpoA* and *secY* on a low copy number plasmid with inducible expression. The parental strain (Additional File 1: Table S1, Parental) and the two most affected movants, S10Tnp-1120 and S10TnpC2+479, were transformed with either of these plasmids or the empty vector. Next, the μ of the transformed strains was determined through automated growth curves. If lower RNAP and/or translocon activity was involved in the observed phenotypes, growth rate differences between the parental and movant strains should lessen or disappear upon *rpoA* and *secY* overexpression. Results on Additional File 1: Fig. S2 show that the growth rate was significantly lower in the movants compared to the parental strain independently of the genes expressed on the plasmid carried. Since the plasmids expressing *rpoA* or *secY* did not rescue the growth defect, the impact of S10 relocation on cell physiology results from dosage reduction of RP genes within the locus.

### Transcriptome analysis of the movant strain set

The physiological effects of S10 relocation are due to dosage reduction of RP genes. Changes in translation were not enough to explain the observed physiological effects. Hence, we reasoned that alternative mechanisms must be involved. To detect genes whose transcription was affected by S10 relocation and search for metabolic pathways responding to RP dosage alterations, we characterized the full transcriptome of the following: S10Tnp-35, the movant in which S10 was slightly moved presenting no phenotype, and the physiologically impaired strains S10Tnp-510, S10Tnp-1120, and S10TnpC2+479 (Fig. 1b). We collected the samples in fast-growing conditions during exponential phase ensuring maximal S10 dosage differences, and then, we compared each movant’s transcriptome to the one of the parental strain.

**Fig. 3:**
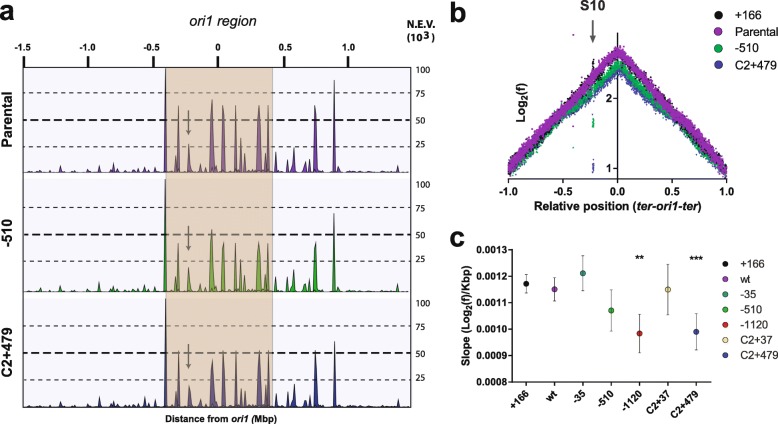
Genome-wide transcription and replication activity along the genome. **a** Transcriptional activity across Chr1. RNA-seq reads were mapped along the Chr1 of *V. cholerae*. The histograms represent mapped read normalized to the genome-wide total volume along both replichores in *ter1-ori1-ter1* order. Normalized expression values (NEV) are shown along the distance from *ori1* in megabase pair which is shown on top. Each graph represents one strain: parental (purple), S10Tnp-510 (green), and S10TnpC2+479 (blue). The plots of the whole strain set are in Fig. S4. The 400-Kbp flanking *ori1* are highlighted in orange. The arrow indicates the peak corresponding to the S10 locus. **b** MFA profiles are obtained by plotting the Log_2_ frequency of reads (normalized against reads from a stationary phase of a parental strain control) at each position in the genome as a function of the relative position on the *V. cholerae* main chromosome with respect to *ori1* (to reflect the bidirectional DNA replication) using 1000-bp windows. Results for the parental (purple), the S10Tnp+166 (black), the S10Tnp-510 (green), and the S10TnpC2+479 (blue) movants show their differences in read coverage. The arrow highlights the S10 position in the abscissa, reflecting dosage alterations. **c** S10 relocation effect on replication dynamics was quantified by averaging the obtained slope for each replichore for at least 4 independent MFA experiments in fast-growing conditions. Results are expressed as the mean slope with 95% CI. Statistical significance was analyzed by one-way ANOVA two-tailed test. Then, Tukey’s test was done to compare the mean values obtained for each strain. Statistically different slopes are indicated as follows: **p < 0.01 and ***p < 0.001

We first looked at the read coverage along the chromosomes, a parameter accounting for the genome-wide transcriptional activity. Surprisingly, we observed that the transcription of the *ori1* region slightly decreased as a function of the distance between S10 and *ori1* (Fig. 3a and Additional file 1 Fig. S3 and Table S3).

### Replication dynamics are altered in the most affected movants

Given that a specific mechanism regulating the expression of such a wide genomic region seems unlikely, we wondered if the change in the expression of *ori1* region was linked to changes in global replication pattern. To assess this, we studied the replication dynamics of the genome of the whole strain set using marker frequency analysis (MFA) in fast-growing conditions. For this, we aligned genomic DNA reads from exponentially growing cells of each strain to the *V. cholerae* genome. For each replicon, there is a linear relationship between the Log_2_ number of reads covering the locus and its genomic position between the *oriC* and the *ter* [36] (Fig. 3b). This allows for robust quantification of replication dynamics across the bacterial genome with unprecedented resolution of replication fork speed and the *ori* and *ter* region locations [25, 36–38]. To better quantify these differences, we calculated the average slope (Log_2_(frequency)/Kbp) along both replichores, which estimates the replication speed for each strain (Fig. 3c). MFA analysis revealed significant differences in replication dynamics across the strain set. The parental strain, the S10Tnp+166, and the S10Tnp-35 displayed a similar slope (Additional file 1: Table S4). Conversely, the most affected movants, S10Tnp-1120 and S10TnpC2+479, where S10 was relocated at the termini of Chr1 and Chr2, showed a significantly lower slope (*p* < 0.01, Fig. 3b, c and Table S4). S10Tnp-510 and S10TnpC2+37 displayed an intermediate value not significantly different from either group. Coincidentally, the calculated slope closely correlated to the S10 locus genomic position (*r* = − 0.78, *p* < 0.05), its dosage (*r* = 0.8, *p* < 0.05), the ori1/ter1 ratio (*r* = 0.91, *p* < 0.005), and μ (*r* = 0.9, *p* < 0.01) (Additional file 1: Fig. S5). This suggests that the genomic location of S10 impacts DNA replication activity, slowing down replication when S10 is far from *ori1*. These data (Fig. 3b, c and Additional file 1: Table S4) indicate that DNA coverage decreases at the *ori1* region with increasing *ori1*-S10 distance matching the changes in transcriptional coverage observed in RNA-seq data.

### Differentially expressed genes upon S10 relocation

We next analyzed the transcriptomic data to find which genes and pathways differentially transcribed with respect to the parental strain in S10Tnp-35 and in the affected movants S10Tnp-510, S10Tnp-1120, and S10TnpC2+479 (Fig. 1b, c).

**Fig. 4:**
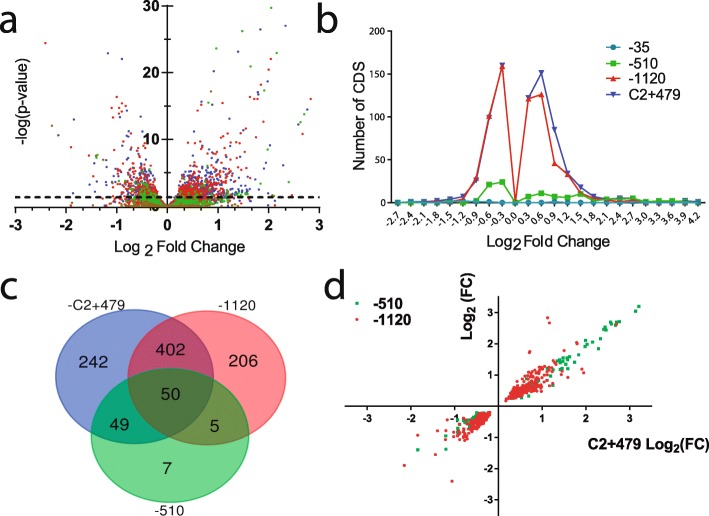
S10 relocation impacts gene expression genome-wide in a distance-dependent manner. **a** Volcano plot displaying differentially expressed genes in S10Tnp-35 (brown), S10Tnp-510 (green), S10Tnp-1120 (red), and S10TnpC2+479 (blue). Horizontal dotted line shows *p* = 0.05. **b** The number of coding sequences (CDS) as a function of Log_2_(FC) of strains S10Tnp-35 (turquoise), S10Tnp-510 (green), S10Tnp-1120 (red), and S10TnpC2+479 (blue). **c** Venn diagram displaying shared genes between S10Tnp-510 (green), S10Tnp-1120 (red), and S10TnpC2+479 (blue). **d** Expression correlation between movant strains. Dots correspond to individual CDS. The Log_2_(FC) of each gene in S10Tnp-510 (green) or S10Tnp-1120 (red) was plotted as a function of Log_2_(FC) in S10TnpC2+479

Using volcano plots, we analyzed the statistical significance of the changes in transcription of each gene (− Log_10_(*p* value)) as a function of its transcriptional Log_2_ of fold change (Log_2_ (FC)) compared to the parental strain. We observed more transcriptionally altered genes with higher distances between the S10 locus and *ori1* (Fig. 4a). S10Tnp-35, a strain presenting no phenotype used as a control of the neutrality of the relocation process, displayed only 8 genes with significant (*p* < 0.05) transcriptional change (Table 1, Additional file 2: Dataset S1). S10Tnp-510, displaying a ~ 7% growth rate reduction (Fig. 1c), showed 111 genes with significantly altered transcription (Table 1, Fig. 4a, Additional file 2: Data Set S1). Finally, the most affected movants, S10Tnp-1120 and S10TnpC2+479, displayed a transcriptional change in 664 and 742 genes. This represents 17.95% and 20.06% of their full gene repertoire. Most of altered genes in the movants were upregulated (Fig. 4b and Table 1). These transcriptional perturbations were relatively small in magnitude since only a 26%, a 10.8%, and a 14.15% of altered genes presented alterations greater than 2-fold in S10Tnp-510, S10Tnp-1120, and S10TnpC2+479, respectively. Meanwhile, upregulated genes showed 2.8-fold, 1.6-fold, and 1.7-fold average increases, respectively (Table 1, Fig. 4b, and Additional file 2: Data Set S1). In the three movants, the downregulated genes displayed a smaller perturbation of ~ 0.7-fold (Table 1).

**Table 1:** Quantitative and qualitative expression changes in the movant strains

	-35	-510	-1120	C2+479
Number of upregulated genes	2 (1)	62 (37)	361 (64)	439 (88)
Mean upregulation^a^	n/d	1.5 ± 0.97	0.67 ± 0.41	0.78 ± 0.56
Number of downregulated genes	6 (4)	49 (2)	301 (9)	303 (17)
Mean downregulation^a^	n/d	− 0.5 ± 0.24	− 0.49 ± 0.26	− 0.52 ± 0.29
Total number of altered genes	8	111 (39)	662 (72)	742 (105)
Altered functions	–	E, P, V	E, O, R, V, N	E, O, R, V, N, F, P, U

The number of differentially expressed genes (*p* < 0.05) compared to parental strain in fast-growing conditions. The number in parenthesis represents genes whose expression varies more than 2-fold. The magnitude of expression change is quantified as the average of the Log_2_(FC) ± standard deviation

^a^Average of the Log_2_(FC) ± standard deviation. *n/d* not determined

The differentially expressed gene profile is similar in S10Tnp-510, S10Tnp-1120, and S10TnpC2+479 movants since a large fraction of transcriptionally altered genes in a movant were also regulated in either of the other two movants (Fig. 4c, Additional file 1: Table S5). Shared genes showed similar levels of transcriptional change across the movants (Fig. 4d and Additional file 1: Table S5). For example, the degree of change in altered genes of S10Tnp-510 and S10Tnp-1120 was highly correlated (*r* = 0.927, *p* < 10^−24^). The differentially expressed genes were not confined to specific chromosome regions nor associated to a specific replicon: S10 relocation produced homogeneously distributed changes in *V. cholerae* gene transcription (Additional file 1: Fig. S6).

To identify the functions or metabolic pathways altered by S10 relocation, we classified *V. cholerae* genes in 25 functional categories using the eggNOG database v.4.0 [39] (Additional file 1: Supp. Text). We then identified the categories with over- or under-representation of genes with altered transcription levels in S10Tnp-510, S10Tnp-1120, and S10TnpC2+479 with respect to the full repertoire of *V. cholerae* genome (Additional file 1: Table S6, Fig. S7; Additional file 2: Data Sets 2 and 3).

Genes from the category “Translation, ribosomal structure, and biogenesis” (J) were not significantly altered, which is consistent with the results above showing that S10 relocation did not alter the translation capacity (Fig. 2). The category “Amino acid transport and metabolism” (E) was statistically altered in all three movants. The category “Posttranslational modification, protein turnover, chaperones” (O) was the most affected category in S10Tnp-1120 and S10TnpC2+479, since about 65% of its genes showed higher transcription in the movants (Additional file 1: Table S6, Additional file 2: Data Set S3). The list of upregulated genes within this category was dominated by chaperones and heat-shock proteins. Strikingly, the highest transcriptional changes occurred in the main pathway for cytosolic protein folding [40]: *grpE* (VC0854), *dnaKJ* (VC0855-6), and both copies of the *groEL-groES* system (VC2664-5 and VCA0819-20). Many transcriptionally altered genes were involved in protein export and ion transport, belonging to several significantly perturbed categories (Table 1 and Additional file 2: Data Set S3). Based on the analysis of functional categories, we observed that *V. cholerae* responds to S10 relocation by altering amino acid synthesis pathways, by increasing the transcription of chaperones and proteases probably to degrade misfolded proteins, and by activating the expression of transporters and permeases.

### Cytoplasm is more fluid in the most affected movants

During exponential growth, ribosomes account for up to 30% of bacterial dry weight [41]. S10 encodes half of the ribosomal proteins, very highly expressed constituting more than a third of total *E. coli* proteins [30]. Therefore, it is likely that a reduction in S10 expression results in macromolecular crowding alterations as observed in other systems [42, 43]. Macromolecular crowding is crucially important in biochemical reactions; however, how it impacts cellular physiology remains mostly unexplored [26–28]. It is well documented that it influences protein folding and aggregation and perturbs protein-nucleic acid interactions [44]. On the other hand, DNA replication has an absolute dependence on macromolecular crowding [43, 45]. Therefore, the reduction in replication fork dynamics (Fig. 3b, c) and the alteration of genes linked to protein folding, protein degradation, permeases, and transport systems (Table 1 and Additional file 2: Data Set S3) observed upon S10 relocation can be interpreted in light of changes in macromolecular crowding caused by a lower RP concentration.

To test this hypothesis, we measured the viscosity of the cytoplasm in the parental strain and in the most affected movants, S10Tnp-1120 and S10TnpC2+479. We expected a more viscous cytoplasm in the parental strain since it expresses S10 genes at higher levels generating a greater concentration of RPs than the movant strains. Differences in cytoplasm viscosity can be uncovered by FRAP experiments on GFP expressing strains. For this, the fluorescence recovery time is measured after bleaching a part of the bacterial cytoplasm [46, 47]. Since the small size and the comma shape of *V. cholerae* complicate the procedure, we generated elongated cells by deleting the Chr2 replication-triggering site (*crt*S) [25] in cells expressing GFP (Additional file 1: Table S1). These mutants present a defective replication of the secondary chromosome. Therefore, S10TnpC2+479 should have even less copies of S10 per cell and, concomitantly, display higher cytoplasmic fluidity than S10Tnp-1120. The elongated phenotype allows photobleaching part of the cytoplasm.

**Fig. 5:**
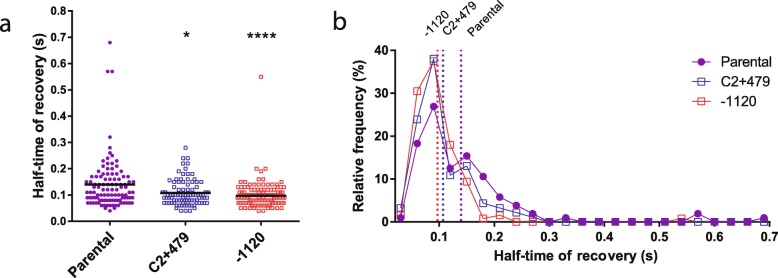
S10 relocation impacts cytoplasm fluidity. **a** Half-time of fluorescence recovery (τ) in the parental-1120 (purple, *n* = 104) and the most affected movants S10Tnp-1120 (red, *n* = 128) and S10TnpC2+479 (blue, *n* = 92) in a gfpmut3* Δ*crtS* genetic context after bleaching part of the cytoplasm. The line indicates the mean τ value, and each dot indicates the obtained value for a cell. Statistical significance was analyzed using the Kruskal-Wallis non-parametric tests followed by Dunn’s multiple comparisons using parental as control respectively. *p < 0.05; ****p < 0.0001. **b** Histogram showing the relative frequency of τ to observe the distribution of the values. The vertical dotted line shows the mean value as in **a**

In the Δ*crtS* context, the parental strain displayed a significantly longer half-time recovery of fluorescence (τ) than the movants (Fig. 5a, Additional file 1: Supp. Text). The collected data showed a high dispersion due to biological variability; however, τ distribution was different in the movants when compared to the parental strain (Fig. 5b) which displayed a τ of 139.7 ms (95% confidence interval (CI) 120.4–158.9 ms; median = 110 ms; *n* = 104). As expected, S10Tnp-1120 showed a τ of 97.3 ms (95% CI 88.31–106.3 ms; median = 90 ms; *n* = 128), significantly shorter than the parental strain (*p* < 0.0001). S10TnpC2+479 displayed a τ of 107.5 ms (95% CI 97.39–117.52 ms; median = 100 ms; *n* = 92), statistically lower than the parental strain (*p* < 0.05) but not significantly different from S10Tnp-1120. The more fluid cytoplasm in the movants could be a consequence of fewer S10-encoded RP suggesting that S10 relocation far from *ori1* reduces cytoplasm macromolecular crowding.

### Growth rate and replication dynamics alterations in movants are alleviated in hyperosmotic conditions

**Fig. 6:**
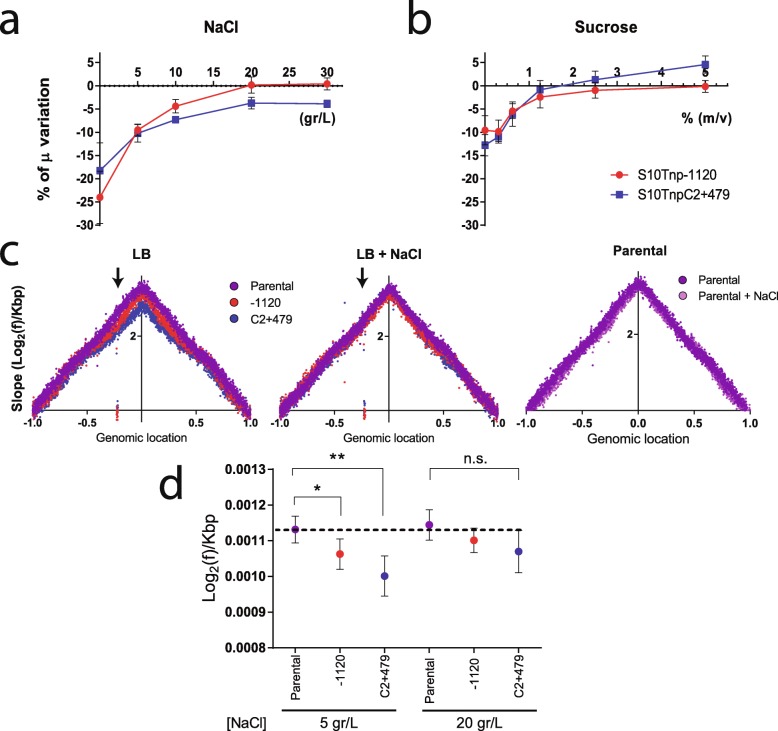
S10 relocation effects are reduced in hyperosmotic conditions. **a** Growth rates of the parental and the indicated movant strains in LB with increasing NaCl concentrations were quantified by averaging the obtained μ in 6 independent experiments with at least 3 biological replicates. The growth of each movant was normalized to the μ of the parental strain, and the percentage of the variation (μ %) ± SEM with respect to parental strains is shown as a function of NaCl concentration of growth medium. **b** Changes in growth of the movant strains with respect to parental strain are shown as a function of sucrose concentration. Data was treated as in **a**, but results correspond to 4 independent experiments with at least 3 biological replicates. **c** MFA profiles are plotted as in Fig. 3b. Results for the parental (purple), the S10Tnp-1120 (red), and the S10TnpC2+479 (blue) strains in LB in the presence of 5 g/L (LB, left panel) or 20 g/L (LB + NaCl, center panel) are shown. The arrow highlights the S10 position in the abscissa, reflecting S10 dosage alterations. The right panel corresponds to MFA of the parental strain when NaCl concentration is 5 or 20 g/L (LB or LB + NaCl). **d** Replication dynamics in the presence of 5 or 20 g/L of NaCl assessed by calculating the slope for each replichore for 2 independent MFA experiments. Dots indicate mean ± SD. Statistical significance was analyzed by one-way ANOVA two-tailed test and Tukey’s test for multiple comparisons. Significance is indicated as follows: n.s., non-significant; *p < 0.05 and **p < 0.01

In line with lower macromolecular crowding, we observed a reduction in cytoplasm viscosity in the movants. To test the possible impact of such molecular crowding alterations on the physiology of the movants, we employed an osmotic stress approach [48–50]. This consists of subjecting strains to a hyperosmotic environment. In these culture conditions, water exits the cell reducing the macromolecular crowding differences between the strains. Therefore, growth differences between the parental strain and the movants should be reduced with increasing solute concentration. To test this, we performed automated growth curves in rich media with increasing NaCl concentrations, comparing the μ of the parental strain to S10Tnp-1120 and S10TnpC2+479 movants. As depicted in Fig. 6a, growth rate differences between the parental strain and the movants were reduced as NaCl concentration increased. Since this phenomenon could be explained by the nature of the solute of choice (e.g., putative differential sensitivity to NaCl), we repeated these assays using sucrose as an alternative compound. As shown in Fig. 6b, results were very similar, suggesting that this phenomenon depends on osmotic changes and cannot be attributed to the nature of the solute (see also Additional file 1: Fig. S9).

Notably, the μ of the parental strain was not significantly reduced in the range of 5 to 20 g/L NaCl (Additional file 1: Fig. S8). Meanwhile, the growth of movants varied significantly along this concentration range, displaying a reduced growth at 5 g/L and 10 g/L and reaching its maximum at 20 g/L (Additional file 1: Fig. S8). Consequently, growth differences observed are not due to impairment of the parental strain in hyperosmotic conditions. Beyond this concentration growth rate is impaired in all strains, probably due to hyperosmotic stress (Additional file 1: Fig. S8, 30 g/L). We conclude that μ differences caused by S10 relocation far from *ori1* can be counterbalanced by artificially increasing cytoplasmic crowding.

Upon S10 relocation far from *ori1*, we observed a lower replication coverage in the movants suggesting that DNA replication activity diminished, suggesting a lower replication speed in the movants (Fig. 3c). Since molecular crowding is crucial for chromosome replication [43, 45], we used the osmotic stress approach to test if the observed replication dynamics defects in movants could be compensated. For this, we performed MFA analyses of the parental strain and the S10Tnp-1120 and S10TnpC2+479 movants in the presence of 5 or 20 g/L of NaCl. In these culture conditions, the parental μ is unaffected. In contrast, movant strains grew ~ 12% slower than the parental strain but they were able to rescue the growth defect at higher NaCl concentrations (Fig. 6a and Additional file 1: Fig. S8). Importantly, a concentration of 20 g/L of this solute increased external osmolality without impacting general physiology. We avoided higher NaCl concentrations that could lead to pleiotropic effects (Additional file 1: Fig. S8). Indeed, the addition of the solute had no effect on the replication dynamics of the parental strain (Fig. 6c, right panel, and 6d). As in earlier experiments, MFA analyses revealed that the movants have a significantly lower slope than the parental strain. Increasing NaCl concentration to 20 g/L made their slopes converge diminishing replication dynamics differences (Fig. 6c, d, Additional file 1: Fig. S10). The integration of these and the previous observations suggests that lower expression of RP caused by S10 relocation (Fig. 1b) leads to lower macromolecular crowding (Fig. 5), which negatively impacts replication (Fig. 3b). This fits the observation that addition of external NaCl, causing water loss and thus narrowing differences in macromolecular crowding, leads to similar replication dynamics between the parental and the movant strains (Fig. 6d, Additional file 1: Fig. S10).

## Discussion

Comparative genomics suggests that gene order coordinates cell cycle to the expression of key functions necessary for cellular homeostasis [4, 11, 19, 20], but few papers provided experimental support [13, 14, 51]. A notable case is that of ribosomal genes which are located near the *oriC* in fast-growing bacteria [19, 20]. By systematically relocating S10, the main cluster of RP genes (Fig. 1c), we proved that its genomic location determines its dosage and expression in *V. cholerae* (Fig. 1b). S10 repositioning far from *ori1* leads to larger generation times, lower fitness, and less infectivity [22, 23]. These effects are dependent on S10 dosage. However, the mechanism explaining how RP dosage affects cell physiology was still missing.

The most straightforward explanation was that high RP dosage due to multifork replication increases their expression maximizing protein biosynthesis capacity [19, 20]. Our initial hypothesis was that movants in which S10 was far from *ori1* would have a lower translation capacity, easily explaining lower growth and fitness of these movants. Surprisingly, we found that in the most affected movants, translation capacity reduction could not explain the observed physiological changes (Fig. 2). We do not rule out that translation impairment may have an effect in the cellular physiology; however, it must have a secondary role in the phenotypes displayed in the affected movants. Slight differences in protein production between the parental strain and the most affected movants could only be detected when measuring maximum protein synthesis capacity (Additional file 1: Fig. S1). All strains showed similar sensitivity to ribosome-targeted antibiotics, suggesting similar ribosome numbers (Fig. 2c). The movants displayed a larger proportion of assembled ribosomal subunits; hence, more free ribosomal subunits are engaged in translation in the movant strain, compensating putative deficiencies in the translation apparatus (Fig. 2e). Interestingly, the S10TnpC2+479 displayed a small peak of ~ 21s that might correspond to precursors of 30s subunit typically associated to cells displaying ribosome assembly deficiencies [52]. Meanwhile, complementation of movants with *secY* and *rpoA*, two S10 genes not related to ribosome biogenesis, failed to rescue the growth defect demonstrating the relevance of RP in the observed phenotype. In sum, although dosage reduction of S10-encoded RP genes caused the observed phenotypes, it is unlikely that this is a consequence of translation defects.

Deep sequencing techniques revealed less transcriptional activity in the region flanking *ori1* (Fig. 3a) and lower replication velocity in the most affected movants (Figs. 3b, c and 6c). Since highly expressed genes that account for a large majority of transcriptional activity in the cell (i.e., *rrn*, RP genes) cluster at this chromosomal region, slight changes in its dosage may globally impact cell physiology [4, 11] and may be responsible for the slight reduction in translational activity observed (Additional file 1: Fig. S1). Meanwhile, differential expression analysis revealed that the transcriptional response is not limited to the *ori1* region (Fig. S6), and encompasses a large number of genes that show slightly but consistently altered transcription in the most affected movants (Fig. 4). Furthermore, the number of these genes increases with distance between S10 and *ori1* (Table 1, Fig. 4 a, b and S6). The latter observation corresponds to biologically meaningful transcriptional changes since furthest relocations caused larger perturbations (Fig. 4a, b), and the majority of altered genes were common to the different movants (Fig. 4c), where they showed similar transcriptional changes (Fig. 4d). This strongly suggests the presence of a common mechanism that slightly affects gene expression at a large scale. Amino acid metabolism and transport genes were less transcribed while there was an upregulation of genes helping protein folding and cellular transporters (Table S5, Additional file 2: Data sets S1 and S3). Importantly, and in line with previous data (Fig. 2), the transcription of translation genes seems to be unaffected in the movants reinforcing the notion that lower protein biosynthesis capacity was not enough to explain the physiological alterations that we observed.

Molecular crowding has a well-known key role in biochemical reactions. Even if its impact on physiological processes has been poorly studied [28], two processes—DNA replication and protein folding—are strongly influenced by macromolecular crowding [29]. Since the discovery of DNA replication, the presence of crowding agents such as polyethylene glycol was shown to be absolutely necessary to reproduce DNA polymerase activity in vitro [43, 45]. In parallel, macromolecular crowding greatly impacts protein aggregation and folding [29], although the in vivo consequences of how the latter occurs are still a matter of debate [44, 53]. It was recently shown that ribosomes are important contributors of macromolecular crowding in the cytoplasm both in prokaryotic and eukaryotic systems [42, 43]. All this information leads us to suggest that upon S10 relocation, the consequent fewer RP may lead to homeocrowding [26] perturbations. To the best of our knowledge, this is the first study exploring the consequences of lower macromolecular crowding conditions since most works linking this physicochemical factor to physiology focus on situations of increased crowding [43, 54, 55]. Concomitantly, we observed reduced replication activity (Fig. 3c), as well as induction of proteases and chaperones to cope with protein aggregation and misfolding (Table 1 and Additional file 1: Fig. S6). Notably, in the most affected movants, the genes coding for the three main chaperone systems—*grpE*, *dnaKJ*, and *groEL-groES* [40]—were among the most strongly induced. The lower transcription of protein and ion transporters could be used for intracellular environment restoration (Table S4, Additional file 1: Fig. S6) and could be a natural consequence of the change in cytoplasm osmotic pressure. We next tested experimentally if S10 relocation could alter homeocrowding. First, using FRAP, we observed slight but statistically significant alterations in the fluidity of the cytoplasm of the most affected movants compared to the parental strain (Fig. 5 a, b, Additional file 1: Supplementary Text). This supports the notion that lower expression of RP associated with movants lowers cytoplasm macromolecular crowding. In the *ΔcrtS* context, we did not detect differences in cytoplasmic fluidity between the S10Tnp-1120 and S10TnpC2+479 movants, expected from lower S10 copy number in the latter by Chr2 loss. We believe that the detrimental effects of *crtS* deletion [25] can explain this. In the S10TnpC2+479 movant, S10 dosage reduction enhances fitness loss, as reflected by slower growth and the presence of small non-viable cells in the microscope not further analyzed (data not shown). When Chr2 replication is inhibited, the fusion of both chromosomes—mainly between their terminal regions—occurs at relatively high frequency [56]. Therefore, the S10TnpC2+479 *ΔcrtS* population might in part consist of cells with fused chromosomes. In this scenario, S10 dosage would not decrease below 1 copy per cell.

The osmotic stress approach provided strong evidence supporting the notion that S10 dosage deficit perturbs cellular homeocrowding. In rich medium, movant strains grow slower than the parental strain. With increasing solute concentrations, this growth deficit is reduced (Fig. 6a, b). In the case of NaCl, the parental strain grew normally in the range from 5 to 20 g/L (Additional file 1: Fig. S8). Outside of this range, growth rate was reduced. Growth was particularly impaired at concentrations below 5 g/L where culture development was very variable due to hyposmotic stress (Fig. 6a and data not shown). Interestingly, movants looked more sensitive than the parental strain to lower solute concentrations. We think that movants express less ribosomal proteins which account for a large fraction of the bacterial proteome, which in turn constitutes a large proportion of the cytoplasmic macromolecules [57]. It is known that about 0.5 g of water is bound per gram of cytoplasmic macromolecules [48, 58]. Therefore, movants may lose their capacity to retain water, suffering from a situation similar to being exposed to hyposmotic conditions. Meanwhile, the μ of the parental and the movants was similar when exposed to 20 g/L or beyond. All strains suffered from physiological alteration beyond this concentration since at 30 g/L of NaCl they displayed a growth impairment compared to the 20 g/L, suggesting that detrimental hyperosmotic conditions altered the strains similarly.

Recent work shows that specific ribosomal protein genes link cell growth to replication in *Bacillus subtilis* [59]. We observed similar effects since S10 dosage correlated growth rate and *oriC*-firing frequency (Fig. 3b, c, S6 and Table S3). In the cited study, the authors attribute this effect to ribosomal function. Although in our system, the effects were milder, we do not rule out the possibility that S10 relocation alters cellular physiology through a reduction in protein synthesis. But this effect is unlikely to account for the full magnitude of the observed phenotypes (Fig. 2) especially as it is relieved in hyperosmotic conditions. We believe that this could be due to a number of factors including the following: (i) the many regulatory mechanisms that control ribosomal protein expression at the translation level, which could partially compensate transcription reduction; (ii) the fact that ribosomal subunits are found in excess with respect to assembled ribosomes; (iii) the possibility that an eventual reduction in functional ribosomes can be compensated by faster translation rates [60–62]; and (iv) finally, it has been described, particularly in *Vibrio* sp*.* CCUG 15956 [63], that ribosomes are available in excess of numbers needed for exponential growth. Such large ribosome quantities would have been selected as an ecological survival strategy that allows for fast growth restoration after its arrest in rapidly changing environmental conditions [64]. Hence, lower S10 expression could be buffered at many levels and protein production might be only mildly impacted. Molecular crowding reduction might however not be as easily compensated. Therefore, movant strains possess a less crowded cytoplasm where DNA polymerase activity is reduced and more chaperones are needed. This would embody a novel mechanism which could explain how ribosomal protein gene position influences growth rate.

## Conclusions

The order of key genes along the chromosomal *ori-ter* axis is phylogenetically conserved in bacteria [11]. However, its influence in cell physiology and its role in genome evolution remain unclear. The chromosomal position of RP genes is baised towards *oriC*, particularly in fast-growing bacteria [20]. A very reasonable explanation for this observation is that such positional tendency allows recruiting multifork replication to enhance RP expression and ribosome biogenesis during exponential growth. Indeed, bacterial growth closely correlates to ribosomal protein content. This has been attributed to the role ribosomes have in protein synthesis [65, 66]. We propose that on top of that, ribosome concentration may change the macromolecular crowding conditions to optimize biochemical reactions, in particular in protein folding and DNA replication [28, 29]. We provide evidence indicating that this is the case for replication dynamics in *V. cholerae*. Our experiments suggest that the genomic position of S10 contributes to generate the RP levels necessary to attain optimal cytoplasmic macromolecular crowding. Besides connecting ribosomal gene position to growth in *V. cholerae*, this mechanism could link ribosome biogenesis to cell cycle in bacteria. During exponential phase, when RP production is maximal and ribosomes represent 30% of cell weight, crowding peaks. This leads to the highest *oriC*-firing frequency. Upon nutrient exhaustion, ribosome production is reduced and the cytoplasm macromolecular crowding diminishes, slowing down replisome dynamics.

## Materials and methods

### General procedures

Genomic DNA was extracted using the GeneJET Genomic DNA Purification Kit while plasmid DNA was extracted using the GeneJET Plasmid Miniprep Kit (Thermo Scientific). PCR assays were performed using Phusion High-Fidelity PCR Master Mix (Thermo Scientific). Strains and plasmids used in this study are listed in Table S1.

### Culture conditions

For fast-growing conditions, bacterial cultures were done in Lysogeny Broth Lennox formulation (LB) at 37 °C with maximum agitation. For harvesting cells in fast-growing conditions, ~ 30 μL of an ON culture was used to inoculate pre-warmed 250 mL Erlenmeyer flasks with 70 mL of LB and agitation was set to 250 rpm. For selection, the following antibiotic concentrations were used: chloramphenicol (3 μg/mL), kanamycin (25 μg/mL), spectinomycin (100 μg/mL), carbenicillin (50 μg/mL), and zeocin (25 μg/mL). NaCl and sucrose were added at the indicated concentrations. Strains and plasmids used are listed in Table S1. For strains expressing *secY* and *rpoA* from pBAD43, cells were cultured in LB (for leak expression), in LB supplemented with 1% glucose (expression repression), or in LB with 0.2% arabinose (maximum induction).

### Automated growth curve measurements

Automated growth curves were performed in 96-well plates avoiding the use of external rows and columns. ON cultures were diluted 1/1000 in LB. Bacterial preparations were distributed at least by triplicate in p96 microplates. Growth curve experiments were performed using a TECAN Infinite Sunrise microplate reader (Thermo), following the OD_600nm_ every 5 min at 37 °C on maximum agitation. Growth rate was obtained using a custom Python script coupled to the Growthrates program [67].

### Protein production capacity

For estimating GFP production, we performed V. cholerae gfpmut3* automated growth curves in a TECAN Infinite 200 microplate reader (Thermo), following OD_600nm_ and GFP fluorescence over time. Data was analyzed using GraphPad Prism 6. For flow cytometry, strains were grown in fast-growing conditions until early exponential phase (OD_450_ ~ 0.2). Then, 50 μL was diluted in 800 μL of PBS. The fluorescence of 20.000 events was recorded in a MACSQuant 10 analyzer (Miltenyi Biotec). Cells were detected using Side Scatter Chanel (SSC) in Log_10_ scale. Data analysis was done using the Flowing Software 2.5.1 (www.flowingsoftware.com). For luciferase activity measurement, *Vibrio cholerae::RL* strains were cultured until OD_450nm_ ~ 0.2. For each experiment, three samples of 20 μL were harvested and directly measured using the Renilla Luciferase Assay System (Promega).

### MIC determination

The MICs of Gm, Cm, and Er were determined using E-test® and the disk diffusion method following the manufacturer’s instructions (Biomérieux).

### Ribosome profiling

Ribosomal 70s, 50s, and 30s species from the indicated *V. cholerae* strains were isolated as previously described [68, 69]. Early exponential phase cultures (OD_450nm_ ~ 0.2) were harvested by centrifugation. Subsequent steps were performed at 4 °C. The pellet was resuspended in ice-cold buffer A (20 mM HEPES pH 7.5, 50 mM NH4Cl, 10 mM MgCl2, 5 mM β-mercaptoethanol, 0.1 mM PMSF) in the presence of Ribolock (Thermo Fisher Scientific). DNase I was added up to 2 μg/mL and kept for 20 min at 4 °C. Cells were lysed by two passes at 11,000–15,000 psi using Emulsiflex. Cell debris were removed by two centrifugation steps at 30,000*g* for 30 min. Then, 0.8 mL of cold 60% sucrose buffer A was added to RNAse-free 5 mL ultraclean tubes for ultracentrifugation in a SW55Ti (Beckman). The ribosome-containing supernatant was used to fill these tubes, and an ultracentrifugation step was performed for 16 h at 150,000*g*. Ribosomes were recovered from the bottom 0.8 mL of 60% sucrose buffer A and dialyzed using a Float-a-lyzer G2 in buffer A. Sedimentation velocity was determined in a Beckman XL-I Analytical Ultracentrifuge. Double sector quartz cells were loaded with 400 μL of buffer A as reference and 380 μL of sample (3 μm), and data were collected at 120,000 rpm from 5.8 to 7.3 cm using a step size of 0.003 cm without averaging. Sedimentation velocity data were analyzed using the continuous size-distribution model employing the program SEDFIT.

### RNA preparation and sequencing for transcriptomic studies

RNA was prepared as described in [70]. We performed four independent biological replicates for each sample. Briefly, 20 mL of an early exponential phase culture was recovered by centrifugation at 4500 rpm for 10 min at 4 °C. Then, RNA was extracted using TRIzol (Thermo Fisher Scientific). Residual DNA was removed with TURBO DNAse (Ambion). RNA quality (total, depleted, and purified) was checked on the Bioanalyzer 2100 (Agilent). Samples were checked for RNA integrity number > 8. The rRNA was depleted using the MicrobExpress kit (Ambion), and libraries were built using the TruSeq Stranded RNA LT Sample Prep Kit (Illumina) and checked for concentration and quality on Bioanalyzer and QuBit (Invitrogen). Sequencing of multiplexed libraries was performed on a HiSeq 2500 (Illumina). Then, in-house quality control process was applied to reads that passed the Illumina quality filters (raw reads). The sequences of the Illumina adapters and primers used during the library construction were removed from the whole reads. Low-quality nucleotides were removed from both ends. Trimmed reads were aligned to the *V. cholerae* reference genome using Bowtie [71] with default parameters. Aligned reads were counted using HTSeq Count [72]. Further quality control and differential expression analysis was performed using methods described above [73–75]. Graphics were done using the GraphPad software, specific online service for Venn diagram (http://bioinformatics.psb.ugent.be/webtools/Venn/) and Circos Plot [76]. The sequence data was submitted to the GenBank Sequence Read Archive (SRA) (see above). Accession numbers for these samples are SRR8316520, SRR8316521, SRR8316528, SRR8316529, SRR8316526, SRR8316527, SRR8316524, SRR8316525, SRR8316522, SRR8316523, SRR8316530, SRR8316531, SRR8316518, SRR8316519, SRR8316516, SRR8316517, SRR8316514, SRR8316515, SRR8316512, and SRR8316513.

### RNA-seq statistical analysis

Count data were analyzed using R version 3.1.2 [77] and the Bioconductor package DESeq2 version 1.6.1 [73]. Data were normalized with DESeq2 and the “shorth” parameter. The dispersion estimation and statistical test for differential expression were performed with default parameters (including outlier detection and independent filtering). The generalized linear model was set with strain (parental, S10Tnp-1120, S10Tnp-35, S10Tnp-510, and S10TnpC2+479 levels) as main effect. Since samples were prepared 4 times independently, the date of sample preparation was also included into the model as a blocking factor to catch more variability and increase the statistical power. Raw *p* values were adjusted for multiple testing according to the Benjamini and Hochsberg (BH) procedure [74], and genes with an adjusted *p* value lower than 0.05 were considered differentially expressed.

### Whole chromosome transcriptional activity comparisons

Reads were mapped as previously described [78] to a custom assembled linear version of the *V. cholerae* that starts (base 0) at the *ter* and finishes at the *ter*, with the *ori1* at the center of the sequence. Total reads mapped to this sequence were counted and normalized as previously described [78]. Fold changes were calculated using normalized values, and *p* values were calculated as previously described [78].

### Marker frequency analysis, slope, and ori1/ter1 ratio calculation

Genomic DNA extracted from early exponential phase (OD_450 nm_ ~ 0.15) was used for library preparation using a PCR-free protocol. Libraries were sequenced on an Illumina MiSeq sequencer using 100- to 150-base-length paired-end reads for 100× genome coverage. The resulting trimmed FastQ files were analyzed using R2R script to obtain the frequency of each locus along the genome, removing repeated sequences [23, 25, 36]. Then, the Log_2_ frequencies every 1000-bp window were then plotted as a function of their relative position on chromosome 1 in *ter1-ori1-ter1* order. Slopes were obtained from linear regression of plots of Log_2_ frequencies along replichore length from ter1 to ori1 (*R*^2^ > 0.95). The slopes represent the Log_2_ frequency change per kilobase pair. The frequency of ori1 and ter1 was quantified by averaging 50 frequency data points corresponding to ori1 and ter1 zones. The S10 frequency was calculated by averaging panels corresponding to VC2569 and VC2599, respectively. These values were used to calculate S10 dosage by calculating the S10/ter1 ratio. The sequence data was submitted to GenBank SRA under the following accession numbers: SRR11398735, SRR11398734, SRR11398726, SRR11398731, SRR11398733, SRR11398727, SRR11398728, SRR11398732, SRR11398729, SRR11398730, SRR11396016, SRR11396017, SRR11396015, SRR11396018, SRR11396019, SRR11396014, SRR11396020, SRR11393293, SRR11393294, SRR11392757, SRR11392758, SRR11392762, SRR11392760, SRR11392754, SRR11392756, SRR11392755, SRR11392763, SRR11392752, SRR11392753, SRR11392759, SRR11392761, SRR11365216, SRR11365215, SRR11365217, SRR11365218, SRR11365214, SRR11365132, SRR11365130, SRR11365133, SRR11365131, SRR11365134, and SRR11363959.

### Functional characterization of the transcriptomic response

*V. cholerae* N16961 genes were aligned against the eggNOG database v.4.0 [39]. Only hits with at least 50% similarity and *e* value < 0.05 were used. Each protein was assigned to the best functional category, according to the percentage of similarity and the length of the alignment. We then calculated the fraction of categories enriched in the fraction of differentially expressed genes, compared to abundances of the different eggNOG categories in the *V. cholerae* genome. The over- or under-representation of protein families was assessed statistically using the Pearson chi-square test with the Benjamini-Hochberg correction for multiple test. For further validation, this test was performed 10,000 times in random subsamples of 30% of the differentially expressed genes.

### FRAP

For measurement of GFP synthesis, stationary phase cultures of *V. cholerae* strains were diluted 1/300 in fresh LB. Then, 6 μL was distributed on an LB agar pad within a Gene Frame (Thermo Fisher) and covered with a cover slip. When indicated, the agar pad was supplemented with Cm at MIC. Cells were then visualized and recorded in a Spinning-Disk UltraView VOX (Perkin-Elmer) equipped with two Hamamatsu EM-CCD (ImageEM X2) cameras. Photobleaching was done using 5–20% of laser power.

For long-term experiments (GFP re-synthesis), detection images were taken every 2 s after photobleaching the total area of the cell for at least 5 min using 200–500 ms of acquisition time. Image analysis was done using ImageJ following photobleached and non-bleached cells in time. The average signal of not-photobleached cells was subtracted to the signal of bleached cells to take into account the decay produced by cell imaging.

For measurement of GFP diffusion within bacteria, we used the exposure times 20 ms at maximum acquisition frame rate after photobleaching a part of the cell area. Then, these movies were analyzed using a specific Jython script developed during the Image Processing School Pilsen 2009 and updated to modern Fiji as described (ImageJ: Analyze FRAP movies with a Jython script, https://imagej.net/Analyze_FRAP_movies_with_a_Jython_script. Accessed 14 August 2019). For every cell analyzed cell length, the photobleached area and the total cell area were determined. Also, a control area was measured. Then, the Jython script was executed. For data analysis, we only kept cells shorter than 6 μm. We only registered half-time values when the function fitted with *R*^2^ > 0.8 (see Additional file 1, Supp. Text, Additional Analysis of FRAP images for further details).

## Supplementary information

**Additional file 1: Figure S1.** The most affected movants, display lower GFP production than the Parental strain at the single cell level. FRAP experiments were performed in LB at 37 °C taking a photo every 2 seconds for at least 5 minutes using the Parental-1120 strain (Parental, violet), the S10Tnp-1120 (red) and S10TnpC2+479 (blue) movants. The parental was also tested in presence of chloramphenicol at MIC (+ Cm). a) A representative plot showing the recovery of fluorescence over time in individual cells. b) The percentage of FRAP at the endpoint of the experiment is shown for all cells tested. Mean with 95% CI is shown. Statistical significance was analyzed by Kruskal-Wallis test (*p*<0.0001). Then Dunn multiple comparison test was made for mean rank obtained for each strain. Letters denote groups being statistically different. **Figure S2.***rpoA* and *secY* overexpression does not rescue growth rate impairment due to S10 relocation. Effect of empty vector, *rpoA* or *secY* expression was quantified by averaging the slope (μ) obtained using 4 biological replicates for each strain in different induction conditions. Results are expressed as the mean μ ± 95% CI. Statistical significance was analyzed using a two-way ANOVA two tailed test and Tukey test for multiple comparisons (p<0.0001). Independently of culture conditions differences are not statistically significant between strains harboring the empty vector, pASB25 or pASB26. Expression was repressed by supplementing culture media with 1% glucose. Induction was achieved adding L-arabinose up to 0.2%. **Figure S3.** RNA Coverage of Chromosome 1 (Chr1) on the full movant strain set. RNA prepared in exponential phase was Deep-Sequenced as described in Materials and Methods. Reads were mapped along the Chr1 of *V. cholerae* and normalized against the full sequence volume. The graphs show the coverage as Normalized Expression Values (dotted lines indicate 75, 50 and 25e10^3^ NEV) along both replichores of the replicon in *ter1-ori1-ter1* order. Each graph represents one strain: Parental (purple); S10Tnp-35 (cyan); S10Tnp-510 (green); S10Tnp-1120 (red); S10TnpC2+479 (blue). The 400 Kbp flanking ori1 are highlighted in orange. A red arrow indicates the peak corresponding to the S10 locus. The coverage of the *ori1* region and the size of the S10 peak lowers with increasing S10-*ori1* distance (see Table S3). This was not the case for Chr2 where the transcriptional activity of the *ori2* region was similar in all strains. Curiously a small increase of the transcriptional activity of the superintegron [81] was observed in S10Tnp-1120 movant (Fig. S4). **Figure S4.** RNA Coverage of chromosome 2 (Chr2) on selected strains. RNA prepared in fast-growing conditions was subjected to deep sequencing. Reads were mapped along the Chr2 of *V. cholerae*. The graphs show Normalized Expression Values along both replichores of the replicon in *ter2-ori2-ter2* order of the parental and the most affected strains. Each graph represents the coverage along Chr2 length of Parental (purple), S10Tnp-1120 (red) and S10TnpC2+479 (blue). The superintegron [81] is highlighted in red (SI). Interestingly, SI region is overexpressed in the S10Tnp-1120 movant. Scale from the first base is shown above the graphs. **Figure S5.** Replication dynamics closely correlates S10 location, dosage, *ori1* firing and growth rate. a) The slopes obtained from the MFA analyses (white circles, right axis) and the growth rate (black squares, left axis) of each strain were plotted as a function of the S10 genomic location. b) S10 dosage (black triangles, left axis) and ori1/ter1 (white triangles, right axis) ratio from MFA analyses for each strain were graphed as a function of the S10 positioning. c) S10 dosage (red), ori1/ter1 ratio (green) and growth rate (blue) are plotted as a function the slope obtained for each strain in MFA analyses. Linear regression for each variable is shown in dotted lines. The data used for each graphic can be found in Table S3. The obtained correlations and their statistical significance are described in the main text of the article. **Figure S6.** S10 relocation produces homogeneously distributed global changes in *V. cholerae* gene expression. Circos plot of genome-wide expression data from strains S10Tnp-35 (Turquoise), S10Tnp-510 (green), S10Tnp-1120 (red) and S10TnpC2+479 (blue). Upper case represents Chr1 while lower case is Chr2 in ter-ori-ter disposition. The origin of replication of each chromosome is represented as *oriC1* and *oriC2* respectively. From inside to outside: Sense and antisense *V. cholerae* genes are depicted as dark orange and orange boxes, respectively. Blue bars represent RNA-seq read counts per gene (scale 1-200,000). Fold-change expression relative to the parental strain is indicated as a green or red solid line indicating fold-expression differences higher than 1.2 or lower than 0.8, respetcively. Dark red dots indicate –log (*p-*value) of the differential expression analysis. Notably, the abundance of significantly altered genes (red dots) from left to right. **Figure S7.** Manhattan Pot showing statistically altered functions across the movant strain set. The abscissa corresponds to specific COG within the S10Tnp-510 (green), S10Tnp-1120 (red) and S10TnpC2+479(blue). S10Tnp-35 is not included since very few genes are differentially expressed displaying no altered functions. The purple line indicates statistical significance fixing α in 0.05. **Figure S8.** The growth rate of the parental strain, S10Tnp-1120 and S10TnpC2+479 was measured using automated growth curves at different NaCl concentrations in rich medium. The mean μ value with SEM of 5 independent experiments is shown. Statistical significance was analyzed by one-way ANOVA two-tailed test. Then Holm-Sidak test was done to compare the means values obtained for each strain. Letters denote groups being statistically different within strains. Differences between strains within each NaCl concentration are denoted as follows: *, *p*<0.05; **, *p*<0.01; ***, *p*<0.001 and n.s. stands for non-significant. **Figure S9.** The growth rate of the parental strain, S10Tnp-1120 and S10TnpC2+479 was measured using automated growth curves at different concentrations of sucrose in LB. The mean μ value with SD of 4 independent experiments by triplicate is shown. All experiments showed the same trend. Statistical significance was analyzed by two-way ANOVA two-tailed test. Then Holm-Sidak test was done to compare the means values obtained for each strain. Letters denote groups being statistically different within strains. Differences between strains within each sucrose concentration are denoted as follows: *, p<0.05; ****, *p*<0.0001 and n.s. stands for non-significant. **Figure S10.** MFA profiles plotted as in Fig. 3b and Fig. 6c. Results for the parental strain (purple), S10Tnp-1120 (red) and the S10TnpC2+479 (blue) movants in LB in presence of 5 gr/L (light) or 20 gr/L (dark) are shown. The statistical analysis of these experiments is shown in Figure 6d. **Table S1.** Full list of plasmids, bacteria strains used in this study. **Table S2.** Exponential fit of fluorescence (GFP production) as a function of OD_600nm._ Data was adjusted to the equation Y=Y_0_*exp(k*X). **Table S3.** Quantification of genome wide transcriptional activity. We calculated the read coverage of the 400 Kbp flanking *ori1* [78]. **Table S4:** Slope, S10 dosage and ori1/ter1 ratio obtained from MFA analysis and growth rate of the analyzed strain set. **Table S5.** Transcriptionally altered genes are shared between movants and regulated in the same manner. The proportion of altered genes that is also found to be regulated in either of the other two movants is shown in the first column (grey). The percentage is shown in parentheses. The two entry table shows that these altered genes were transcriptionally altered in the same way between the movants. We calculated the Pearson Correlation Test for their Log_2_(FC). The value of the test is shown in green while the corresponding p-value is displayed in orange. **Table S6.** Altered functions upon S10 relocation. Genes within *V. cholerae* genome were classified in functional categories using eggNOG database. The table shows the number of genes whose expression is altered in selected functional categories for each movant train and the total genes in the chromosome belonging to each category. The number in parenthesis represents the % with respect the total number of genes. The functions with no genes or with no alterations are not displayed and can be found in Additional file 2, Data Set S3. The total number in the last row also includes functions not displayed.

**Additional file 2.** Data sets of differentially expressed genes and classification into functional categories.

## Data Availability

All data generated or analyzed during this study are included in this published article, its supplementary information files, and publicly available repositories. The RNA-seq datasets are deposited at the GenBank SRA as BioProject PRJNA509993 [79]. The genomic DNA sequencing data for MFA studies are available at SRA as BioProject PRJNA613768 [80].

## Abbreviations

RP: Ribosomal protein
*oriC*: Origin of replication
S10: *s10-spc-α*
*ter*: Terminal region
Chr1: Main chromosome
Chr2: Secondary chromosome
Kbp: Kilobase pairs
Mbp: Megabase pairs
μ: Growth rate
OD: Optical density
GFP: Green fluorescent protein
RL: *Renilla* luciferase
MIC: Minimum inhibitory concentration
Cm: Chloramphenicol
Gm: Gentamicin
Er: Erythromycin
FRAP: Fluorescence recovery after photobleaching
AUC: Analytical ultracentrifugation
MFA: Marker frequency analysis
*crtS*: Chr2 replication-triggering site
τ: Half-time recovery of fluorescence
