# Supplementary material for: Macromolecular crowding links ribosomal protein gene dosage to growth rate in Vibrio cholerae
Source: BMC Biol. 2020 Apr 29;18:43. doi: 10.1186/s12915-020-00777-5 (PMC7191768; doi:10.1186/s12915-020-00777-5)
Supplement: Supplementary file 1 — Additional file 1: Figure S1. The most affected movants, display lower GFP production than the Parental strain at the single cell level. FRAP experiments were performed in LB at 37 °C taking a photo every 2 seconds for at least 5 minutes using the Parental-1120 strain (Parental, violet), the S10Tnp-1120 (red) and S10TnpC2+479 (blue) movants. The parental was also tested in presence of chloramphenicol at MIC (+ Cm). a) A representative plot showing the recovery of fluorescence over time in individual cells. b) The percentage of FRAP at the endpoint of the experiment is shown for all cells tested. Mean with 95% CI is shown. Statistical significance was analyzed by Kruskal-Wallis test (p<0.0001). Then Dunn multiple comparison test was made for mean rank obtained for each strain. Letters denote groups being statistically different. Figure S2.rpoA and secY overexpression does not rescue growth rate impairment due to S10 relocation. Effect of empty vector, rpoA or secY expression was quantified by averaging the slope (μ) obtained using 4 biological replicates for each strain in different induction conditions. Results are expressed as the mean μ ± 95% CI. Statistical significance was analyzed using a two-way ANOVA two tailed test and Tukey test for multiple comparisons (p<0.0001). Independently of culture conditions differences are not statistically significant between strains harboring the empty vector, pASB25 or pASB26. Expression was repressed by supplementing culture media with 1% glucose. Induction was achieved adding L-arabinose up to 0.2%. Figure S3. RNA Coverage of Chromosome 1 (Chr1) on the full movant strain set. RNA prepared in exponential phase was Deep-Sequenced as described in Materials and Methods. Reads were mapped along the Chr1 of V. cholerae and normalized against the full sequence volume. The graphs show the coverage as Normalized Expression Values (dotted lines indicate 75, 50 and 25e103 NEV) along both replichores of the replicon in ter1-ori1-ter1 ord [file 12915_2020_777_MOESM1_ESM.docx]

Supplemental information to:

**Macromolecular crowding links ribosomal protein gene dosage to growth rate in *Vibrio cholerae*.**

Alfonso Soler-Bistué^1,2^, Sebastián Aguilar-Pierlé^1^, Marc Garcia-Garcerá^3,4^, Marie-Eve Val^1^, Odile Sismeiro^5^, Hugo Varet^5^, Rodrigo Sieira^6^, Evelyne Krin^1^, Ole Skovgaard^7^, Diego J. Comerci^2^, Eduardo P. C. Rocha^3^, and Didier Mazel^1^#

^1^Institut Pasteur, Unité Plasticité du Génome Bactérien, UMR3525, CNRS, Paris, France.

^2^Instituto de Investigaciones Biotecnológicas "Dr. Rodolfo A. Ugalde,", CONICET, Universidad Nacional de San Martín, San Martín, Buenos Aires, Argentina.

^3^Institut Pasteur, Microbial Evolutionary Genomics, Département Génomes et Génétique, Paris, France, Centre National de la Recherche Scientifique UMR3525, Paris, France.

^4^University of Lausanne, Department of Fundamental Microbiology, Quartier SORGE, 1003 Lausanne, Switzerland

^5^Institut Pasteur, Plate-forme Transcriptome et Épigenome, Biomics, Centre d'Innovation et Recherche Technologique (Citech), Paris, France.

^6^Fundación Instituto Leloir, IIBBA-CONICET, Buenos Aires, Argentina.

^7^Department of Science and Environment, Roskilde University, Roskilde, Denmark.

# Lead Contact: mazel@pasteur.fr

**Table S1.** Full list of plasmids, bacteria strains used in this study:

| **Name** | | **Relevant genotype and/or phenotype** | **Reference** |
| --- | --- | --- | --- |
| **Plasmids** | | | |
|  |  | |  |
| pJBA28 | delivery plasmid for mini-Tn5-Km-PA1/04/03-RBSII-*gfpmut3**-T0-T1, Amp^R^ and Kan^R^ | | Andersen et al 1998 |
| pCP20 | pSC101*rep*^TS^ [*flp*] | | Cherepanov et al. 1995 |
| pBAD43 | pACYC184 *oriV*, *araBAD*, Spc^R^ | | Guzman LM et al. 1995 |
| pRL-CMV | Wild type Renilla Luciferase (RL) gene for expression in mammalian cells. Used for RL amplification. | | Promega |
| pGL-2Basic | Wild type Firefly Luciferase (FL) gene for expression in mammalian cells. Used for FL amplification. | | Promega |
| pASB12 | pCR-BluntII-Topo(RL gene linked to lox66-Zeo^R^-lox71 cassette) | | This study |
| pASB13 | pCR-BluntII-Topo(FL gene linked to lox66 Zeo^R^-lox71 cassette) | | This study |
| pASB21 | pCR-BluntII-Topo(*gfpmut3***Not*I fragment-lox66-Zeo^R^-lox71) | | Soler Bistué et al 2017 |
| pASB25 | *rpoA* (VC2571) cloned into pBAD43 for its regulated expression. | | This study |
| pASB26 | *secY* (VC2576) cloned into pBAD43 for its regulated expression. | | This study |
|  |  | |  |
| ***Escherichia coli*** | | | |
| DH5α | | F^-^ *endA1 glnV44 thi-1 recA1 relA1 gyrA96 deoR nupG* Φ80d*lacZ*ΔM15 Δ(*lacZYA-argF*)U169, *hsdR17*(r_K_^-^ m_K_^+^), λ^–^ |  |
| ***Vibrio cholerae*** | | | |
| N16961*ChapRΔlacZ* | | N16961::mTn*7hapR^+^* Δ*lacZ*. Er^S^, Gm^R^ and Cm^S^. | Val et al. 2012 |
| Parental -1120 | | PGB-A192::*attB*’*-lox66-dfrB1-lox71* inserted in the intergenic region between VC1508-VC1509. Er^S^, Gm^R^ and Cm^R^. | Soler-Bistué et al. 2015 |
| S10Tnp+166 | | S10 relocated closer to *oriC1* in the intergenic region between VC2739-VC2740. Er^S^, Gm^R^ and Cm^R^. | Soler-Bistué et al. 2015 |
| S10Tnp-35 | | S10 relocated next to its original location in the intergenic region between VC2536-VC2537. Er^S^, Gm^R^ and Cm^R^. | Soler-Bistué et al. 2015 |
| S10Tnp-510 | | S10 relocated at the middle of the left replichore of chromosome 1 in the intergenic region between VC2075-VC2076. Er^S^, Gm^R^ and Cm^R^. | Soler-Bistué et al. 2015 |
| S10Tnp-1120 | | S10 relocated near the *dif* region of chromosome 1 in the intergenic region VC1508-VC1509. Er^S^, Gm^R^ and Cm^R^. | Soler-Bistué et al. 2015 |
| S10TnpC2+37 | | S10 relocated near the *oriC2* in the intergenic region between VCA0030-VCA0031. Er^S^, Gm^R^ and Cm^R^. | Soler-Bistué et al. 2015 |
| S10TnpC2+479 | | S10 relocated near the *dif* sequence of chromosome 2 in the intergenic region between VCA0543-VCA0544. Er^S^, Gm^R^ and Cm^R^. | Soler-Bistué et al. 2015 |
| S10Md(-1120;C2+479) | | Merodiploid bearing *S10-spc-α* copies at the intergenic sequences of VC1508-VC1509 and VCA0543-VCA0544. Er^S^, Gm^R^ and Cm^R^. | Soler-Bistué et al. 2015 |
| *V.cholerae*::*gfpmut3** | | NotI fragment from pJBA28 (Andersen et al. (1998)) containing promoter P_A1/04/3_, RBSII and *gfpmut3** linked to Zeo^R^ from pASB11 gene was inserted in the intergenic region between VC0696-VC0697. | Soler-Bistué et al. 2017 |
| Parental-1120::*gfpmut3** | | *gfpmut3**-ZeoR cassette was inserted in the intergenic region between VC0696-VC0697 in Parental-1120 strain. | Soler-Bistué et al. 2017 |
| S10Tnp-35::*gfpmut3** | | *gfpmut3**-ZeoR cassette was inserted in the intergenic region between VC0696-VC0697 in S10Tnp-35 strain. | This study |
| S10Tnp-510::*gfpmut3** | | *gfpmut3**-ZeoR cassette was inserted in the intergenic region between VC0696-VC0697 in S10Tnp-510 strain. | This study |
| S10Tnp-1120::*gfpmut3** | | *gfpmut3**-ZeoR cassette was inserted in the intergenic region between VC0696-VC0697 in S10Tnp-1120 strain. | This study |
| S10TnpC2+479::*gfpmut3** | | *gfpmut3**-ZeoR cassette was inserted in the intergenic region between VC0696-VC0697 in S10TnpC2+479 strain. | This study |
| Parental-1120::*RL* | | *RL*-ZeoR cassette from pASB12 was inserted in the intergenic region between VC0696-VC0697 in Parental-1120 strain. | This study |
| S10Tnp-35::*RL* | | *RL*-ZeoR cassette from pASB12 was inserted in the intergenic region between VC0696-VC0697 in S10Tnp-35 strain. | This study |
| S10Tnp-510::*RL* | | *RL*-ZeoR cassette from pASB12 was inserted in the intergenic region between VC0696-VC0697 in S10Tnp-510 strain. | This study |
| S10Tnp-1120::*RL* | | *RL*-ZeoR cassette from pASB12 was inserted in the intergenic region between VC0696-VC0697 in S10Tnp-1120 strain. | This study |
| S10TnpC2+479::*RL* | | *RL*-ZeoR cassette from pASB12 was inserted in the intergenic region between VC0696-VC0697 in S10TnpC2+479 strain. | This study |
| Parental -1120  Δ(*aph,cat*) | | Parental-1120. Kanamycin and chloramphenicol resistance cassettes were deleted using a flipase expressing plasmid. Er^S^, Gm^R^ and Cm^S^. | Soler-Bistué et al 2015 |
| S10Tnp-1120 Δ(*aph,cat*) | | S10 relocated near the *dif* region of chromosome 1. Derived from Parental -1120 Δ(*aph,cat*). Er^S^, Gm^R^ and Cm^S^. | Soler-Bistué et al 2015 |
| Parental-1120 (pBAD43) | | Parental strain bearing empty vector pBAD43. | This study |
| Parental-1120 (pASB25) | | Parental strain bearing plasmid for *rpoA* overexpression. | This study |
| Parental-1120 (pASB26) | | Parental strain bearing plasmid for *secY* overexpression. | This study |
| S10Tnp-1120 (pBAD43) | | Strain whose S10 was relocated close to *ter1* bearing empty vector pBAD43. | This study |
| S10Tnp-1120 (pASB25) | | Strain whose S10 was relocated close to *ter1* bearing plasmid for *rpoA* overexpression. | This study |
| S10Tnp-1120 (pASB26) | | Strain whose S10 was relocated close to *ter1* bearing plasmid for *secY* overexpression. | This study |
| S10TnpC2+479 (pBAD43) | | Strain whose S10 was relocated close to *ter2* bearing empty vector pBAD43. | This study |
| S10TnpC2+479 (pASB25) | | Strain whose S10 was relocated close to *ter2* bearing plasmid for *rpoA* overexpression. | This study |
| S10TnpC2+479 (pASB26) | | Strain whose S10 was relocated close to *ter2* bearing plasmid for *secY* overexpression. | This study |
| Parental-1120::*gfpmut3** Δ*crts* | | The Chr2 replication triggering site (*crt*S) a 150 bp Chr1 sequence (coordinates 817950-818100) was replaced by a rifampicin resistance cassette (*arr2*) as in Val et al. (2016) Parental-1120::*gfpmut3** | This study |
| S10Tnp-1120::*gfpmut3** Δ*crts* | | The *crt*S was replaced by a rifampicin resistance cassette (*arr2*) as in Val et al (2016) in S10Tnp-1120::*gfpmut3** movant. | This study |
| S10TnpC2+479::*gfpmut3** Δ*crts* | | The *crt*S was replaced by a rifampicin resistance cassette (*arr2*) as in Val et al (2016) in S10TnpC2+479::*gfpmut3** movant. | This study |

**Table S2:** Exponential fit of fluorescence (GFP production) as a function of OD_600nm._  Data was adjusted to the equation Y=Y_0_*exp(k*X).

|  | **Parental** | **S10Tnp-35** | **S10Tnp-510** | **S10Tnp-1120** | **S10TnpC2+479** | ***gfpmut3^-^*** |
| --- | --- | --- | --- | --- | --- | --- |
| **Y_0_** | 2493 | 2507 | 2482 | 2474 | 2475 | 2808 |
| **k** | 2.080 | 2.144 | 2.277 | 2.299 | 2.029 | 0.7047 |
| **R^2^** | 0.9985 | 0.9964 | 0.9968 | 0.9984 | 0.9970 | 0.9844 |

**Figure S1: The most affected movants, display lower GFP production than the Parental strain at the single cell level.** FRAP experiments were performed in LB at 37°C taking a photo every 2 seconds for at least 5 minutes using the Parental-1120 strain (Parental, violet), the S10Tnp-1120 (red) and S10TnpC2+479 (blue) movants. The parental was also tested in presence of chloramphenicol at MIC (+ Cm). **a)** A representative plot showing the recovery of fluorescence over time in individual cells. **b)** The percentage of FRAP at the endpoint of the experiment is shown for all cells tested. Mean with 95% CI is shown. Statistical significance was analyzed by Kruskal-Wallis test (p<0.0001). Then Dunn multiple comparison test was made for mean rank obtained for each strain. Letters denote groups being statistically different.


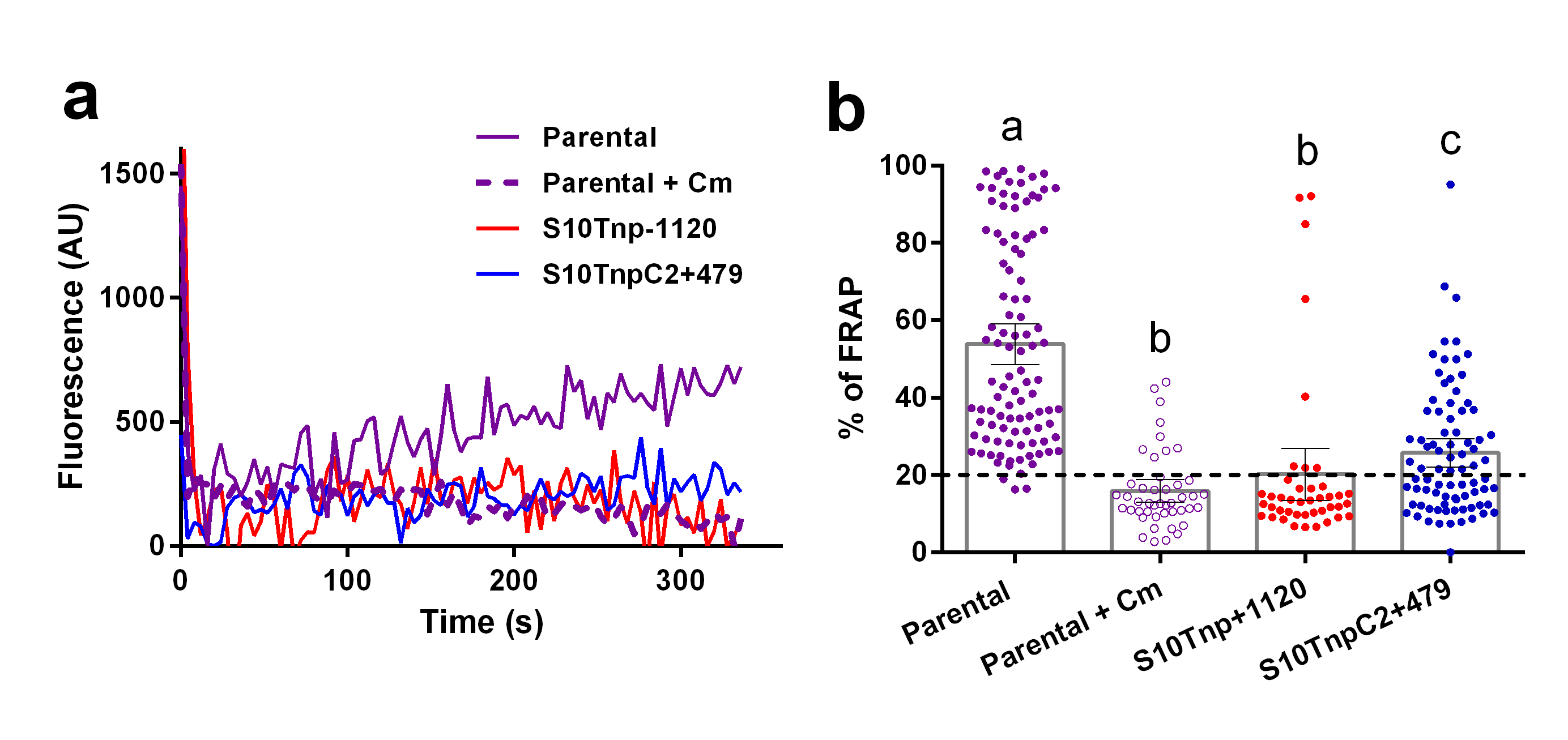


**Figure S2: *rpoA* and *secY* overexpression does not rescue growth rate impairment due to S10 relocation.** Effect of empty vector, *rpoA* or *secY* expression was quantified by averaging the slope (μ) obtained using 4 biological replicates for each strain in different induction conditions. Results are expressed as the mean μ ± 95% CI. Statistical significance was analyzed using a two-way ANOVA two tailed test and Tukey test for multiple comparisons (p<0.0001). Independently of culture conditions differences are not statistically significant between strains harboring the empty vector, pASB25 or pASB26. Expression was repressed by supplementing culture media with 1% glucose. Induction was achieved adding L-arabinose up to 0.2%.

**Figure S3: RNA Coverage of Chromosome 1 (Chr1) on the full movant strain set.** RNA prepared in exponential phase was Deep-Sequenced as described in Materials and Methods. Reads were mapped along the Chr1 of *Vibrio cholerae* and normalized against the full sequence volume. The graphs show the coverage as Normalized Expression Values (dotted lines indicate 75, 50 and 25e10^3^ NEV) along both replichores of the replicon in *ter1-ori1-ter1* order. Each graph represents one strain: Parental (purple); S10Tnp-35 (cyan); S10Tnp-510 (green); S10Tnp-1120 (red); S10TnpC2+479 (blue). The 400 Kbp flanking ori1 are highlighted in orange. A red arrow indicates the peak corresponding to the S10 locus. The coverage of the *ori1* region and the size of the S10 peak lowers with increasing S10-*ori1* distance (see Table S3). This was not the case for Chr2 where the transcriptional activity of the *ori2* region was similar in all strains. Curiously a small increase of the transcriptional activity of the superintegron [1] was observed in S10Tnp-1120 movant (Fig. S4).


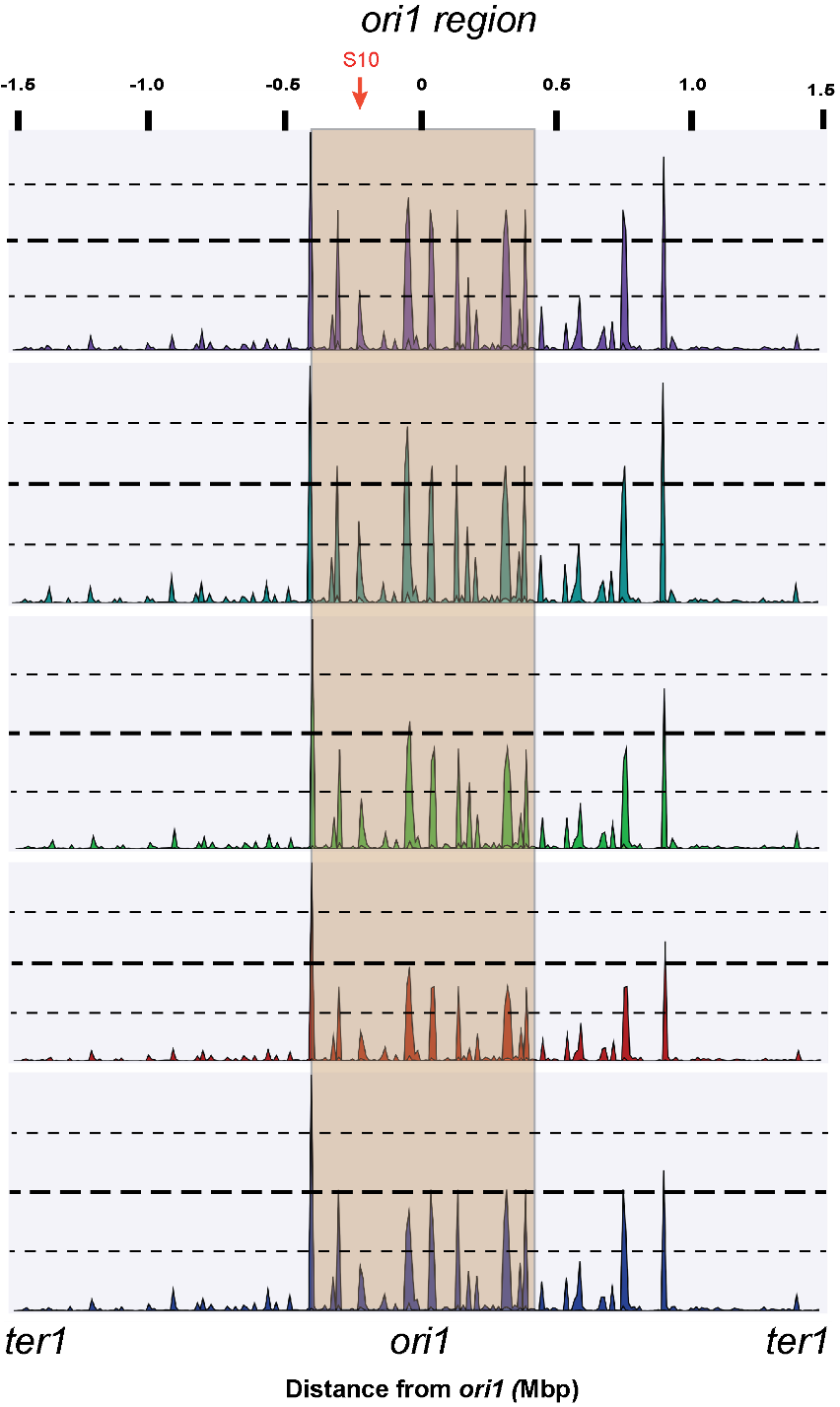


**Figure S4: RNA Coverage of chromosome 2 (Chr2) on selected strains.** RNA prepared in fast-growing conditions was subjected to deep sequencing. Reads were mapped along the Chr2 of *V. cholerae*. The graphs show Normalized Expression Values along both replichores of the replicon in *ter2-ori2-ter2* order of the parental and the most affected strains. Each graph represents the coverage along Chr2 length of Parental (purple), S10Tnp-1120 (red) and S10TnpC2+479 (blue). The superintegron [1] is highlighted in red (SI). Interestingly, SI region is overexpressed in the S10Tnp-1120 movant. Scale from the first base is shown above the graphs.


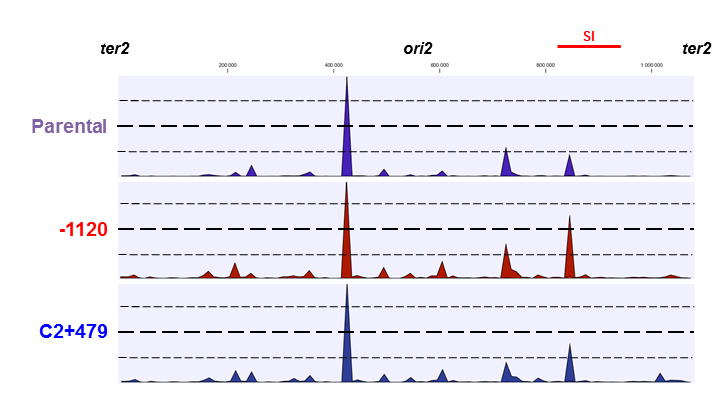


**Table S3: Quantification of genome wide transcriptional activity.** We calculated the read coverage of the 400 Kbp flanking *ori1* [2].

| Movant | FC | p-value |
| --- | --- | --- |
| S10Tnp-35 | **n.s.** | **>0.05** |
| S10Tnp-510 | **-1.042** | **p<10-13** |
| S10Tnp-1120 | **-1.056** | **p<10-25)** |
| S10TnpC2+479 | **-1.044** | **10-8** |

**Table S4:** Slope, S10 dosage and ori1/ter1 ratio obtained from MFA analysis and growth rate of the analyzed strain set.

| **Strain** | **MFA Slope^a^ (Log_2_(f)/Kbp)** | **S10 dosage^a,b^** | **ori1/ter1^a,b^** | **Growth rate^c^** (min^-1^) |
| --- | --- | --- | --- | --- |
| S10Tnp+166 | 1.17 ± 0.042×10^-3^ | 2.94 ± 0.34 | 3.3 ± 0.21 | 0.0182 ± 0.0002 |
| Parental | 1.15 ± 0.07×10^-3^ | 2.54 ± 0.21 | 3.33 ± 0.22 | 0,0184 ± 0.0002 |
| S10Tnp-35 | 1.12 ± 0.041×10^-3^ | 2.72 ± 0.26 | 3.21 ± 0.39 | 0.0182 ± 0.0002 |
| S10Tnp-510 | 1.07 ± 0.09×10^-3^ | 1.76 ± 0.19 | 3.09 ± 0.28 | 0.0172 ± 0.0002 |
| S10Tnp-1120 | 0.98 ± 0.147×10^-3^ | 1.12 ± 0.10 | 2.84 ± 0.49 | 0.0154 ± 0.0004 |
| S10TnpC2+37 | 1.15 ± 0.075×10^-3^ | 1.67 ± 0.06 | 3.22 ± 0.3 | 0.0173 ± 0.0002 |
| S10TnpC2+479 | 0.99 ± 0.09×10^-3^ | 1.16 ± 0.09 | 2.86 ± 0.23 | 0.0151 ± 0.0003 |

**^a^** Expressed as Mean ± SD.

**^b^**The ratio of the frequency of each locus was calculated directly from the MFA data.

**^c^**Data from our former study (Soler-Bistué et al 2015) measured in automated growth curves.

**Figure S5: Replication dynamics closely correlates S10 location, dosage, *ori1* firing and growth rate.**  **a)** The slopes obtained from the MFA analyses (white circles, right axis) and the growth rate (black squares, left axis) of each strain were plotted as a function of the S10 genomic location. **b)** S10 dosage (black triangles, left axis) and ori1/ter1 (white triangles, right axis) ratio from MFA analyses for each strain were graphed as a function of the S10 positioning. **c)** S10 dosage (red), ori1/ter1 ratio (green) and growth rate (blue) are plotted as a function the slope obtained for each strain in MFA analyses. Linear regression for each variable is shown in dotted lines. The data used for each graphic can be found in Table S3. The obtained correlations and their statistical significance are described in the main text of the article.


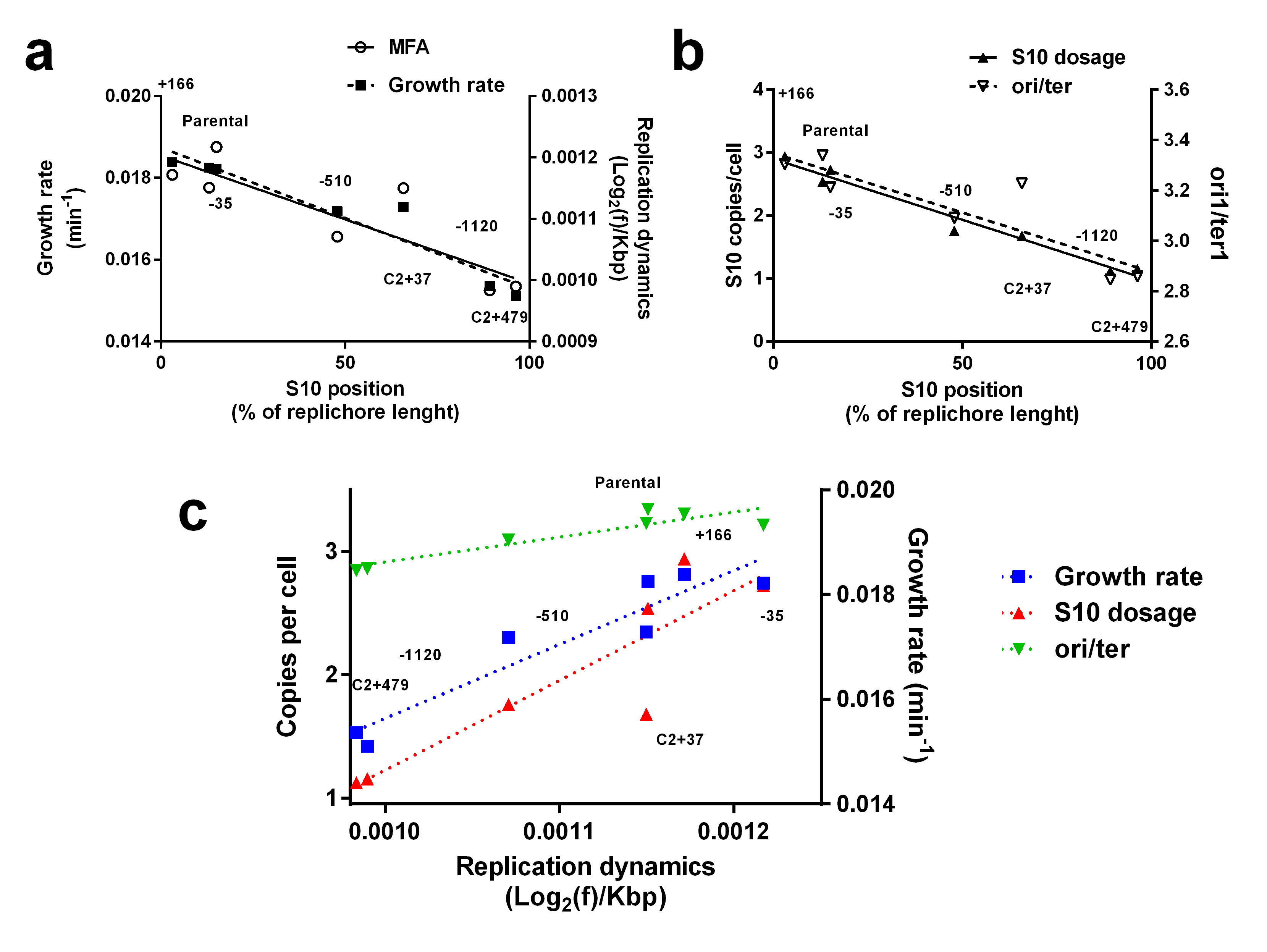


**Table S5: Transcriptionally altered genes are shared between movants and regulated in the same manner.** The proportion of altered genes that is also found to be regulated in either of the other two movants is shown in the first column (grey). The percentage is shown in parentheses. The two entry table shows that these altered genes were transcriptionally altered in the same way between the movants. We calculated the Pearson Correlation Test for their Log_2_(FC). The value of the test is shown in green while the corresponding p-value is displayed in orange.

| **S10Tnp** | **All** | **-510** | **-1120** | **C2+479** |
| --- | --- | --- | --- | --- |
| **-510** | 104/111 (93.7%) |  | 0.927 | 0.992 |
| **-1120** | 457/662(69%) | 10^-24^ |  | 0.946 |
| **C2+479** | 501/742 (67.4%) | 10^-30^ | 10^-33^ |  |

**Figure S6: S10 relocation produces homogeneously distributed global changes in *V. cholerae* gene expression.** Circos plot of genome-wide expression data from strains S10Tnp-35 (Turquoise), S10Tnp-510 (green), S10Tnp-1120 (red) and S10TnpC2+479 (blue). Upper case represents Chr1 while lower case is Chr2 in ter-ori-ter disposition. The origin of replication of each chromosome is represented as *oriC1* and *oriC2* respectively. From inside to outside: Sense and antisense *V. cholerae* genes are depicted as dark orange and orange boxes, respectively. Blue bars represent RNA-seq read counts per gene (scale 1-200,000). Fold-change expression relative to the parental strain is indicated as a green or red solid line indicating fold-expression differences higher than 1.2 or lower than 0.8, respetcively. Dark red dots indicate –log (*p-*value) of the differential expression analysis. Notably, the abundance of significantly altered genes (red dots) from left to right.


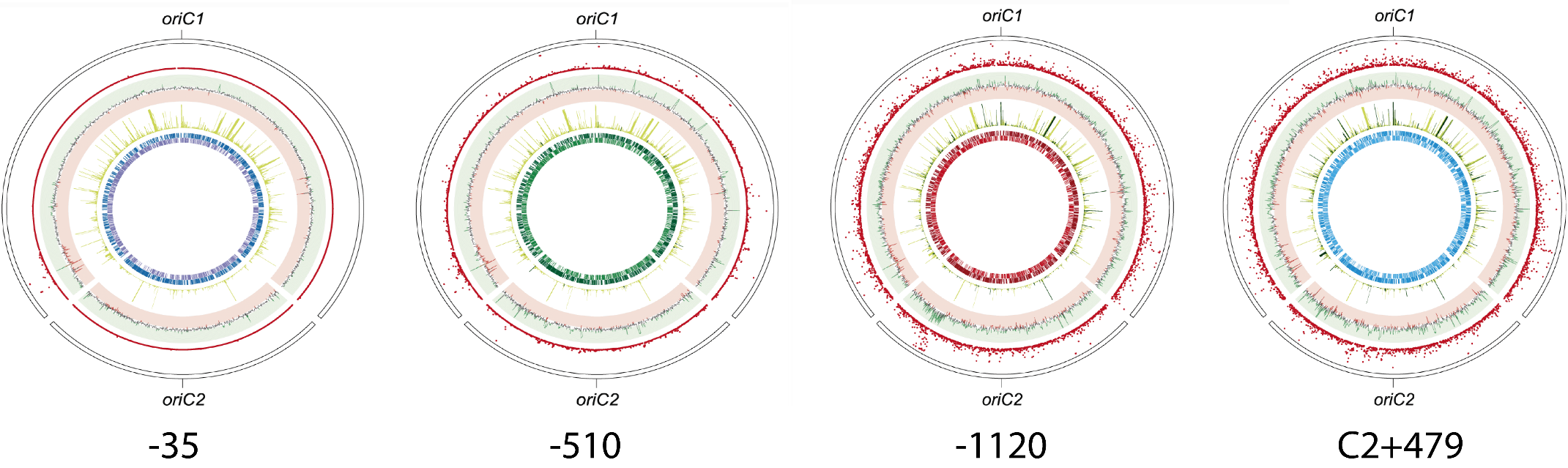


**Table S6: Altered functions upon S10 relocation.** Genes within *V. cholerae* genome were classified in functional categories using eggNOG database. The table shows the number of genes whose expression is altered in selected functional categories for each movant train and the total genes in the chromosome belonging to each category. The number in parenthesis represents the % with respect the total number of genes. The functions with no genes or with no alterations are not displayed and can be found in Additional File 2, Data Set S3. The total number in the last row also includes functions not displayed.

|  | **-510** | | | **-1120** | | | **C2+479** | | | **Total** |
| --- | --- | --- | --- | --- | --- | --- | --- | --- | --- | --- |
|  | **down** | **Up** | **Total** | **down** | **Up** | **Total** | **down** | **Up** | **Total** |  |
| J | 2 | 1 | 3  (2.7) | 10 | 10 | 20 (3.1) | 15 | 11 | 26 (3.6) | **172 (5.06)** |
| V | 0 | 0 | 0 | 5 | 7 | 12  (1.86) | 5 | 8 | 13 (1.79) | **48 (1.41)** |
| U | 1 | 3 | 4  (3.6) | 8 | 9 | 17 | 6 | 9 | 15 | **70 (2.06)** |
| O | 1 | 0 | 1  (0.9) | 14 | 32 | 46 (7.14) | 14 | 26 | 40  (5.51) | **121 (3.56)** |
| E | 16 | 9 | 25 (22.5) | 49 | 24 | 73  (11.3) | 40 | 29 | 69  (9.5) | **236 (6.95)** |
| P | 1 | 26 | 27 (24.3) | 18 | 12 | 30  (4.65) | 15 | 35 | 50 (6.9) | **201 (5.9)** |
| **Total** | **49** | **62** | **111** | **296** | **348** | **644** | **296** | **429** | **725** | **3395** |

**Figure S7: Manhattan Pot showing statistically altered functions across the movant strain set.** The abscissa corresponds to specific COG within the S10Tnp-510 (green), S10Tnp-1120 (red) and S10TnpC2+479(blue). S10Tnp-35 is not included since very few genes are differentially expressed displaying no altered functions. The purple line indicates statistical significance fixing α in 0.05.


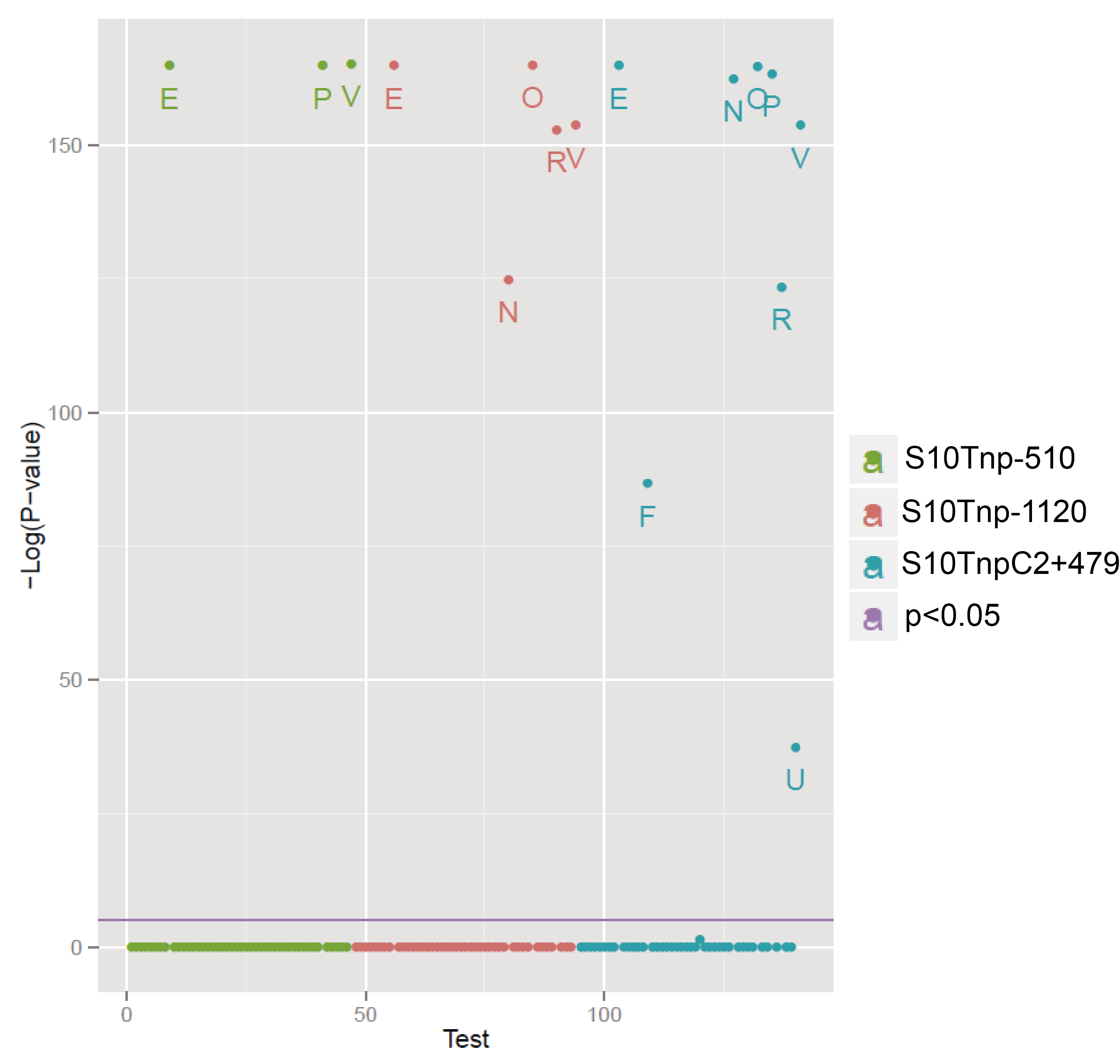


**Figure S8:** The growth rate of the parental strain, S10Tnp-1120 and S10TnpC2+479 was measured using automated growth curves at different NaCl concentrations in rich medium. The mean µ value with SEM of 5 independent experiments is shown. Statistical significance was analyzed by one-way ANOVA two-tailed test. Then Holm-Sidak test was done to compare the means values obtained for each strain. Letters denote groups being statistically different within strains. Differences between strains within each NaCl concentration are denoted as follows: *, p<0.05; **, p<0.01; ***, p<0.001 and n.s. stands for non-significant.

**Figure S9:** The growth rate of the parental strain, S10Tnp-1120 and S10TnpC2+479 was measured using automated growth curves at different concentrations of sucrose in LB. The mean µ value with SD of 4 independent experiments by triplicate is shown. All experiments showed the same trend. Statistical significance was analyzed by two-way ANOVA two-tailed test. Then Holm-Sidak test was done to compare the means values obtained for each strain. Letters denote groups being statistically different within strains. Differences between strains within each sucrose concentration are denoted as follows: *, p<0.05; ****, p<0.0001 and n.s. stands for non-significant.


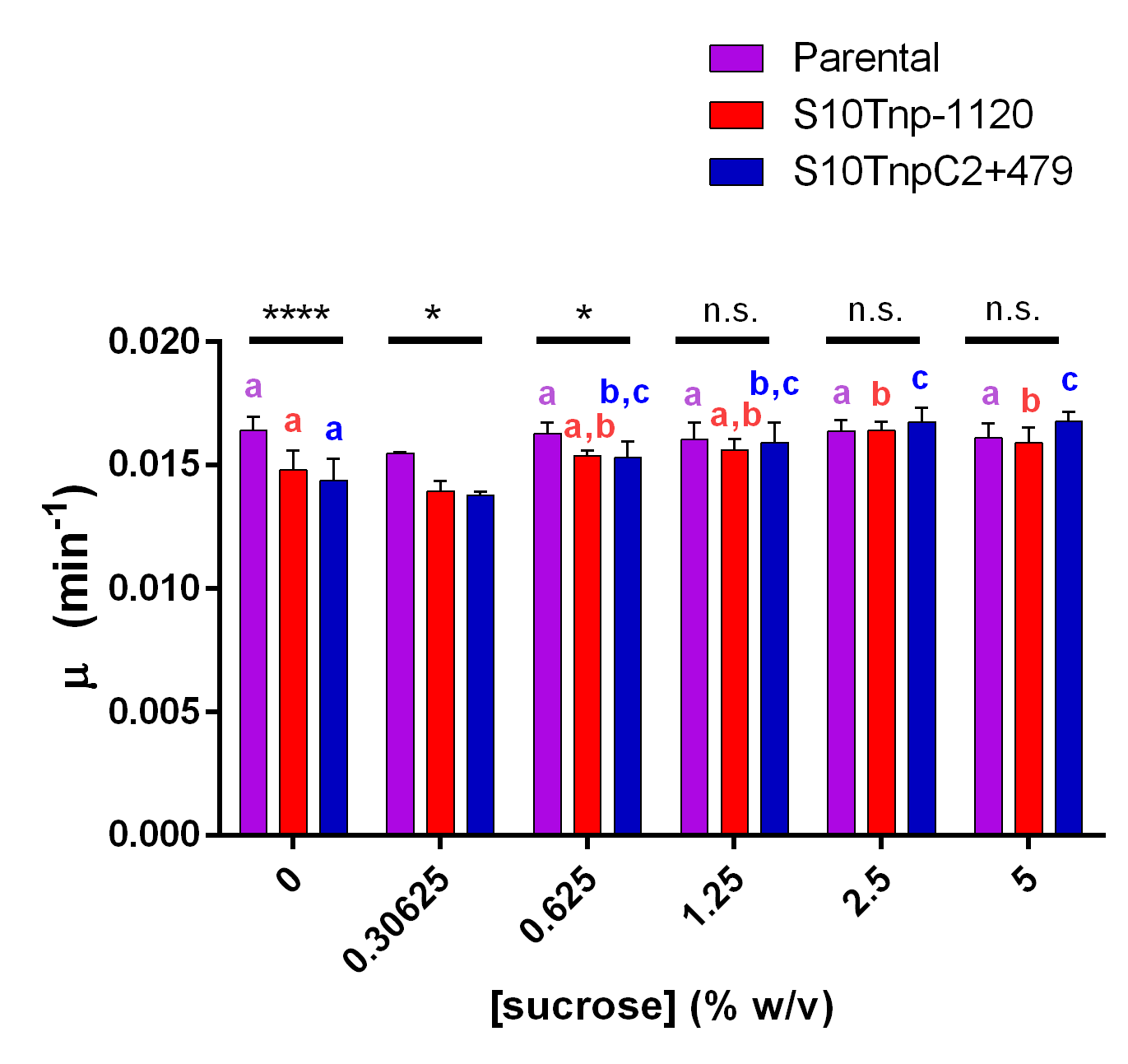


**Figure S10:** MFA profiles plotted as in Fig. 3b and Fig.6c. Results for the parental strain (purple), S10Tnp-1120 (red) and the S10TnpC2+479 (blue) movants in LB in presence of 5 gr/L (light) or 20 rg/L (dark) are shown. The statistical analysis of these experiments is shown in Figure 6d.


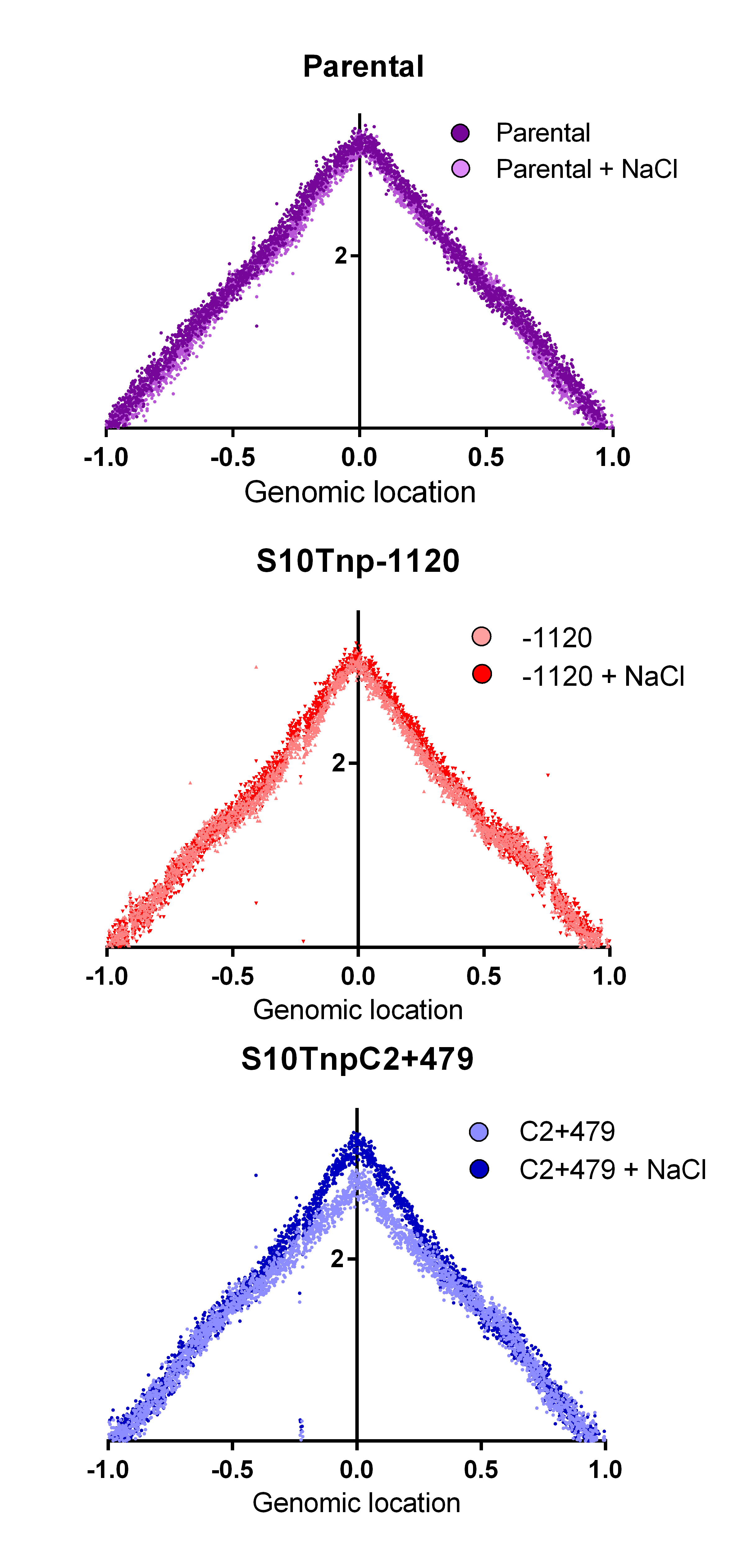


**Supplementary Text**

**Extended methods:**

**COG CATEGORIES:**

INFORMATION STORAGE AND PROCESSING

[J] Translation, ribosomal structure and biogenesis

[A] RNA processing and modification

[K] Transcription

[L] Replication, recombination and repair

[B] Chromatin structure and dynamics

CELLULAR PROCESSES AND SIGNALING

[D] Cell cycle control, cell division, chromosome partitioning

[Y] Nuclear structure

[V] Defense mechanisms

[T] Signal transduction mechanisms

[M] Cell wall/membrane/envelope biogenesis

[N] Cell motility

[Z] Cytoskeleton

[W] Extracellular structures

[U] Intracellular trafficking, secretion, and vesicular transport

[O] Posttranslational modification, protein turnover, chaperones

METABOLISM

[C] Energy production and conversion

[G] Carbohydrate transport and metabolism

[E] Amino acid transport and metabolism

[F] Nucleotide transport and metabolism

[H] Coenzyme transport and metabolism

[I] Lipid transport and metabolism

[P] Inorganic ion transport and metabolism

[Q] Secondary metabolites biosynthesis, transport and catabolism

POORLY CHARACTERIZED

[R] General function prediction only

[S] Function unknown

**Additional Analysis of FRAP images:**

To discard that half-time recovery of fluorescence (τ) differences observed were not due to an unintentionally biased analysis we measured and analyzed cell length, cell area, control area, photobleached area and the mobile fraction. As explained in the text, τ was longer in the parental strain. The length and the area of the cell were significantly lower in C2+479. The photo bleached area was not significantly different among strains although there is a trend for parental strain to present a larger one.

|  | Parental | C2+479 | -1120 |
| --- | --- | --- | --- |
| - (ms) | (120.4-158.9) | (97.39-117.52) | (88.31-106.3) |
| cell lenght (µm) | (4.87-5.25) | (4,35-4,72) | (4,85-5,2) |
| Photobleached area (µm^2^) | (1.74-1.95) | (1,51-1,80) | (1,56-1,75) |
| Control Area (µm^2^) | (1.64-1.9) | (1,46-1,73) | (1,67-1,87) |
| Cell Area (µm^2^) | (4.32-4.73) | (3,93-4,32) | (4,07-4,43) |
| Mobile faction (%) | (22.37-25.98) | (21,25-25,21) | (22,33-25,16) |

We next performed spearman correlation analyses between these variables among these strains. Interestingly, cell length correlated with τ in all 3 strains (r= 0.487, r=0.49 and r=0.399 respectively with p<0.0001 in all cases). The photo bleached area presented small r value (~0.2) that was not statistically significant in the parental strain. Therefore, we consider the influence of the bleached area in the obtained τ very mild or inexistent. Finally, we found that the employed control area did not influence the outcome of the experiments since there was not a correlation between τ and this parameter.
